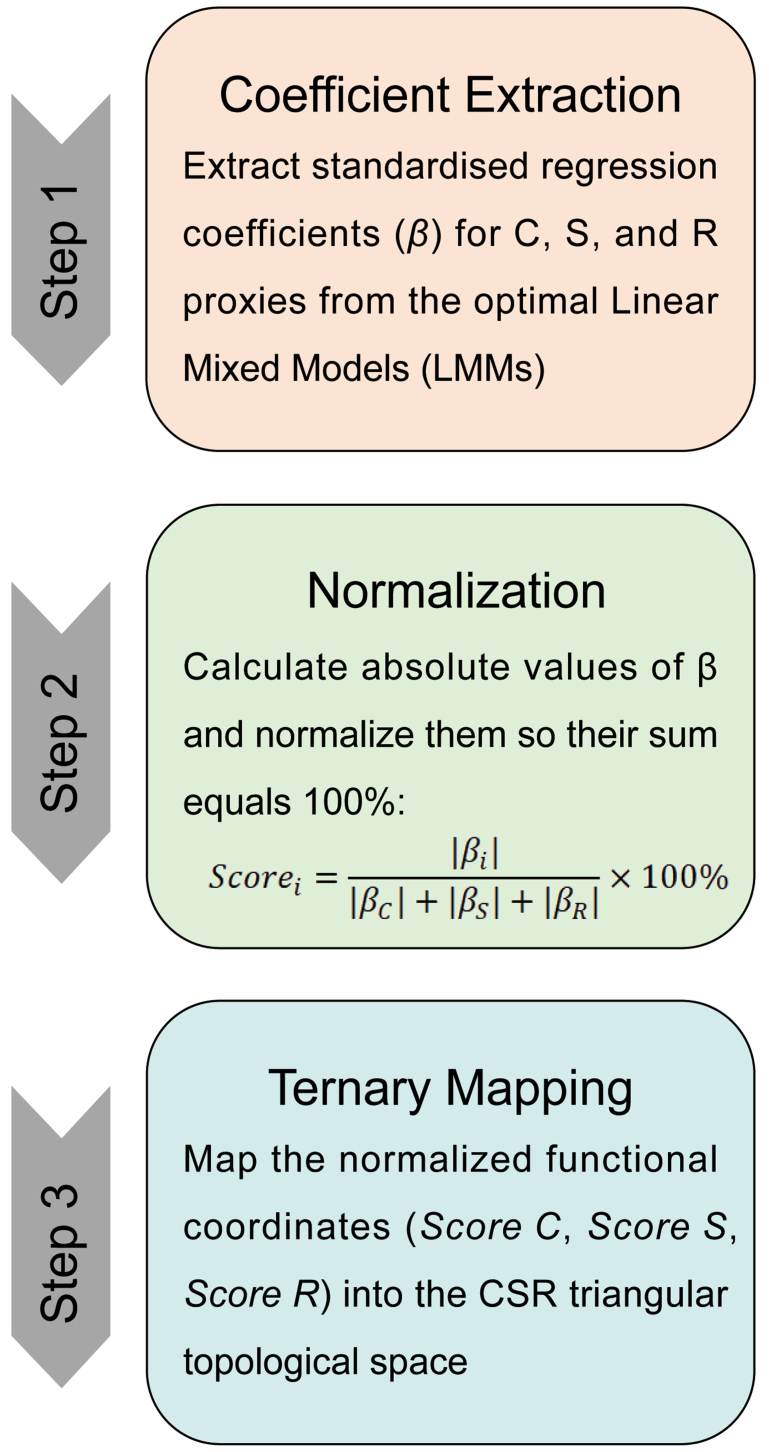# How does plant diversity drive productivity in China’s drylands: Through complementary or mass ratio effects?

**DOI:** 10.1016/j.pld.2026.04.011

**Published:** 2026-05-12

**Authors:** Hao Guo, Hong-Hu Meng, Jin-Fei Yin, Tong Li, Ben-Feng Yin, Xiao-Bing Zhou, Yuan-Ming Zhang

**Affiliations:** a State Key Laboratory of Desert and Oasis Ecology, Key Laboratory of Ecological Safety and Sustainable Development in Arid Lands, Xinjiang Institute of Ecology and Geography, Chinese Academy of Sciences, Urumqi 830011, China; b Xinjiang Key Laboratory of Biodiversity Conservation and Application in Arid Lands, Xinjiang Institute of Ecology and Geography, Chinese Academy of Sciences, Urumqi 830011, China; c Xinjiang Field Scientific Observation Research Station of Tianshan Wild Fruit Forest Ecosystem, Yili Botanical Garden, Xinjiang Institute of Ecology and Geography, Chinese Academy of Sciences, Urumqi 830011, China; d Plant Phylogenetics and Conservation Group, Center for Integrative Conservation, Xishuangbanna Tropical Botanical Garden, Chinese Academy of Sciences, Kunming 650223, China; e School of Life Sciences, Hebei University, Baoding 071002, China

**Keywords:** Biodiversity, Aboveground biomass (AGB), Complementarity effect, Mass ratio effect, CSR strategy, Threshold effect

## Abstract

Dryland biodiversity–productivity relationships remain poorly resolved. Specifically, the environmental conditions governing the shift between complementarity and mass ratio mechanisms remain unclear, limiting the effectiveness of restoration and management strategies. To address this gap, the aim of this study was to investigate the geographical patterns of diversity and biomass production in herbaceous communities along a 2100-km precipitation gradient in North China. We studied how α- and β-diversity affect community-wide productivity using linear mixed-effects models and piecewise structural equation models, along with rolling-window change-point analyses. In arid regions, biomass productivity was primarily driven by interspecific niche complementarity, where higher functional diversity (FD(α)) enhanced resource-use efficiency. However, in semi-arid regions, productivity was regulated by the mass ratio effect, specifically through the traits of dominant species, including community weighted mean height and specific leaf area, as these species exploited broader resource spectra with increasing water availability. A critical mechanistic shift occurred at a mean annual precipitation (MAP) threshold of ∼168 mm (95% CI: 152–171 mm; *p* < 0.001). Below this threshold, productivity was driven by diversity-mediated complementarity and stress tolerant strategies. Conversely, as MAP surpassed 168 mm, the system transitioned to mass ratio control, coincident with a shift toward competitive strategies. Overall, our study provides empirical evidence to guide dryland management: prioritising the maintenance of functional diversity in arid communities, while emphasising dominant-trait optimisation (plant height and specific leaf area) in semi-arid communities to maximise aboveground biomass.

## Highlights

• A 168 mm precipitation threshold separates complementarity and mass ratio effects.
• Functional diversity enhances productivity in arid regions through niche differentiation.
• Dominant trait expression (Plant height, specific leaf area) drives productivity in semi-arid ecosystems.

## Graphical abstract

Contribution of herbaceous plant diversity to community productivity in China’s drylands. Unveiling the shift from complementarity effects in arid regions to mass ratio effects in semi-arid regions.This study investigates the transition processes of ecological drivers regulating aboveground biomass (AGB) along a 2100-km precipitation gradient in northern China. (a) Mechanistic Shift: In arid regions (MAP < ∼168 mm), productivity is primarily driven by the complementarity effect (high functional diversity), enhancing resource use efficiency under stress. A critical threshold occurs at approximately 168 mm, beyond which the mass ratio effect becomes dominant, with productivity increasingly determined by the functional traits (Height, SLA) of dominant species. (b) Strategy Shift: This functional transition corresponds to a shift in community assembly strategies from Stress-tolerant (S) species in water-limited environments to Competitive (C) species in semi-arid regions.

**Figure:**
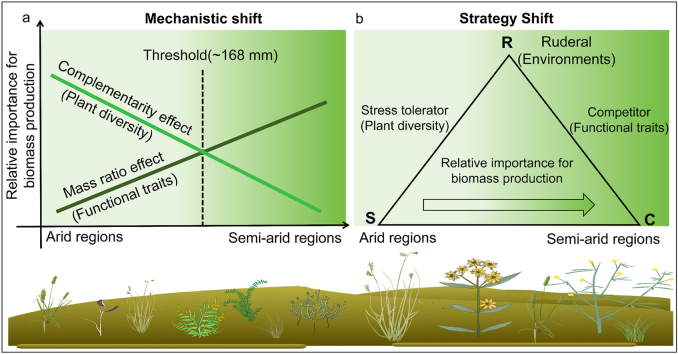

## 1 Introduction

In recent decades, ecological research has focused on the impact of biodiversity on productivity (Grace et al., 2007; Fridley and Grime, 2010; Grossman et al., 2017; Pan et al., 2025). Empirical and theoretical work suggest that biodiversity can enhance biomass production and temporal stability through resource partitioning and compensatory dynamics linked to species turnover (Grime, 1973; Tilman, 1996). This prediction assumes adequate species overlap, providing redundancy and buffering against fluctuations in ecosystem stability. Such buffering may persist despite spatiotemporal variation in species–environment interactions, while niche partitioning can promote higher productivity by alleviating competitive constraints (Río et al., 2017). However, whether these general patterns extend to drylands remains contentious.

Drylands are defined as regions where the aridity index (annual precipitation divided by annual potential evapotranspiration) is below 0.65 (Zomer et al., 2022). Drylands are characterised by chronic resource scarcity and strong rainfall intermittency; consequently, community biomass is often driven by pulse dynamics, episodic recruitment and mortality, and pronounced environmental filtering. These processes can weaken, obscure, or even reverse diversity–productivity relationships depending on the spatial grain and climatic context (Le Bagousse-Pinguet et al., 2019). Indeed, such reported relationships from drylands are inconsistent, varying in direction and magnitude along aridity gradients and among organisational levels (within vs among communities) (Mori, 2018). Although taxonomic, functional, and phylogenetic approaches have been used to link biodiversity with ecosystem functioning (Flynn et al., 2011; Thompson et al., 2015), these facets are often analysed in isolation and at a single scale, posing a challenge in determining whether productivity patterns primarily reflect variation in species richness, shifts in community trait distributions and life-history strategies, or compositional turnover across the landscape. Addressing this gap requires an integrated, multi-faceted evaluation that explicitly quantifies how diversity–productivity linkages vary along continuous precipitation gradients, where ecological transitions may be gradual or abrupt (Mori, 2018).

Examining taxonomic diversity, functional diversity (FD), and phylogenetic diversity across diverse plant groups, ecosystems, and spatial scales is crucial for understanding how biodiversity drives productivity (Loreau and Hector, 2001; Cadotte et al., 2011; Tilman et al., 2014), at both *α* (within-community) and *β* (between-community) levels. The combination of these three metrics provides novel insights that cannot be achieved by focusing on a single metric, as they capture different facets of biodiversity that together reveal complex ecological dynamics. Taxonomic diversity (richness) significantly enhances community productivity (Klironomos et al., 2000; Liu et al., 2015). However, in drylands, this relationship remains especially uncertain because richness can covary with strong environmental filtering and rainfall intermittency. Furthermore, productivity may be disproportionately shaped by shifts in species identity, dominance structure, and compositional turnover, rather than by species numbers alone (Galand et al., 2015; Catano et al., 2023). FD explores the distinct roles or functions of each species (Río et al., 2017), rooted in the concept that functional traits shape a species’ responses to environmental changes. For instance, traits that influence nutrient or water use efficiency are closely linked to a species’ resource competition strategies (Loranger et al., 2013). The trait distributions in drylands are often tightly constrained by abiotic stress. Consequently, functional variation may be compressed within communities while increasing among communities along aridity gradients. This dynamic complicates inference when analyses are restricted to a single organisational level (Venail and Vives, 2013). In contrast, phylogenetic diversity measures the presence of different evolutionary lineages within a community. A phylogenetically diverse community may possess greater adaptability potential due to the broad spectrum of genetic information available (Connolly et al., 2011). Importantly, these facets need not change in parallel in drylands: communities can exhibit similar richness but divergent trait syndromes or lineage composition, or, conversely, display strong turnover without commensurate changes in functional structure.

Understanding the mechanisms linking biodiversity to productivity is central to evaluating ecosystem functioning and associated services (Loreau, 2000; Williams et al., 2017). Importantly, the strength and even the direction of these linkages are unlikely to be invariant across environmental gradients. In drylands, where water availability is chronically limiting and highly intermittent, community assembly and biomass production are strongly shaped by environmental filtering and pulse-driven growth. Therefore, the processes underpinning productivity may change systematically along moisture gradients (Le Bagousse-Pinguet et al., 2019). Under severe water limitation, variation in species’ resource-acquisition strategies and trait differentiation may alter how communities capture and partition scarce resources. Conversely, increasing moisture availability can intensify competitive asymmetries and allow a smaller subset of species to disproportionately determine community-level functioning (Galand et al., 2015). This context dependence suggests that the dominant mechanism linking biodiversity and productivity may shift along precipitation gradients.

Two complementary frameworks formalise these alternative pathways. The mass ratio hypothesis posits that ecosystem functioning is primarily governed by the traits of the most dominant species (ie, those contributing the greatest biomass). In this context, community trait values are commonly characterised using community-weighted means (CWMs) that emphasise dominant functional attributes (Grime, 1998; Díaz et al., 2007; Roscher et al., 2008; Mokany et al., 2008). In contrast, the complementarity hypothesis assumes that co-occurring species differ in resource-use strategies. This differentiation enables a more complete exploitation of available resources and thereby enhances productivity by reducing competition and improving efficiency (Montes et al., 2008; Niklaus et al., 2017). Accordingly, taxonomic, functional, and phylogenetic diversity can be viewed as capturing complementary facets of niche differentiation and turnover. These diverse facets may, in turn, translate into higher community productivity (Kahmen et al., 2006; Mahaut et al., 2019; Amyntas et al., 2023). Framing biodiversity–productivity relationships within these two mechanisms provides a structured basis for testing whether and where dominance-versus complementarity-driven pathways prevail across large-scale moisture gradients.

The Competitiveness, Stress tolerance, and Ruderal (CSR) theory is a pivotal macroecological framework that remains underutilised in examining how biodiversity influences productivity (Grime, 1973; Liao et al., 2021; Mason-Jones et al., 2022). Here, we use the CSR theory as an explicit mechanistic bridge linking community assembly along moisture gradients to the relative importance of complementarity versus mass-ratio effects. Its primary strength lies in encompassing species’ adaptations to competition, stress, and disturbance (Pierce et al., 2017). In relatively mesic portions of the gradient where competition for light and nutrients intensifies, C strategists are expected to dominate biomass and disproportionately determine ecosystem functioning via their acquisitive traits (e.g., greater height). This dominance consequently strengthens mass ratio control via community-weighted CWM trait values (Norris et al., 2011; Wonkka et al., 2013). In contrast, under arid and highly stressful conditions, S strategists with conservative resource-use traits tend to persist, and productivity is constrained by strong environmental filtering. Under these conditions, any positive biodiversity effect is more likely to arise from functional differentiation among coexisting stress tolerant species, consistent with a complementarity pathway (Rosado and Mattos, 2017; Aragon et al., 2023). Moreover, variation across evolutionary lineages can yield non-redundant trait syndromes within CSR space. This variation widens the spectrum of resource acquisition and timing, further supporting complementarity when no single strategy monopolises biomass (Hamilton et al., 2005). Finally, in systems where disturbances and rainfall pulses are prominent, R-strategists (often annuals) can rapidly exploit transient resources, contributing to episodic biomass accumulation and recovery. These rapid responses can potentially modulate the balance between complementarity and mass-ratio effects across time (Bruelheide et al., 2018). Therefore, characterising community CSR composition provides a coherent theoretical framework for anticipating—and testing—mechanistic shifts in biodiversity–productivity relationships along precipitation gradients.

In Northern China, drylands—encompassing arid and semi-arid regions—extend from deserts and desert steppes to meadows and grasslands, covering over 35% of the country’s land area (Meng et al., 2015; Su et al., 2021; Wang et al., 2022a, b). Climate change–driven desertification is increasingly affecting these regions, altering plant diversity and community productivity (Shen et al., 2023). Although these ecosystems have received extensive attention (Maestre et al., 2012; Jing et al., 2015; Zhang et al., 2023; Yao et al., 2025), empirical evidence remains limited on how multiple biodiversity dimensions jointly regulate productivity across broad precipitation gradients. Notably, most large-scale assessments have tended to examine single facets of diversity or a single spatial scale. It remains unresolved whether patterns attributed to “biodiversity effects” primarily reflect within-community richness/trait dispersion, compositional turnover among communities, or their covariance along aridity gradients. Moreover, the intrinsic complexity of drylands (severe water scarcity and nutrient-poor soils) complicates efforts to identify consistent diversity–productivity relationships (Maestre et al., 2012; Berdugo et al., 2020); temporal and spatial variability in rainfall and temperature further confounds these patterns (Delgado-Baquerizo et al., 2017), impeding the establishment of clear causal links. Critically, it remains unclear whether mechanistic controls on productivity shift abruptly at specific precipitation thresholds along the gradient or instead vary continuously, along with how such potential thresholds would differentially structure contributions to *α*-versus *β*-diversity, posing a significant challenge for understanding dryland ecosystems (Maestre et al., 2024).

To address these significant knowledge gaps, we assessed the contribution of herbaceous plant multidimensional diversity to community productivity in the drylands of North China. We explicitly propose two central hypotheses (Fig. 1):

(1) The mechanistic shift hypothesis: the primary driver of biomass production shifts from niche complementarity in arid regions to the mass ratio effect in semi-arid regions. Specifically, we predict that in resource-limited arid environments, productivity depends on high taxonomic and functional diversity to maximise resource use efficiency. Conversely, as environmental filtering relaxes in semi-arid regions, we expect productivity to be governed by the functional traits of dominant species.
(2) The strategic turnover hypothesis: the functional basis of productivity shifts from S strategies in arid regions to C strategies in semi-arid regions. We postulate that the trade-off between survival and growth dictates this pattern. Specifically, arid zones select for persistence traits (S strategy) where competition is low. In contrast, semi-arid regions select for acquisitive traits (C strategy) that capitalise on higher resource availability.

**Fig. 1:**
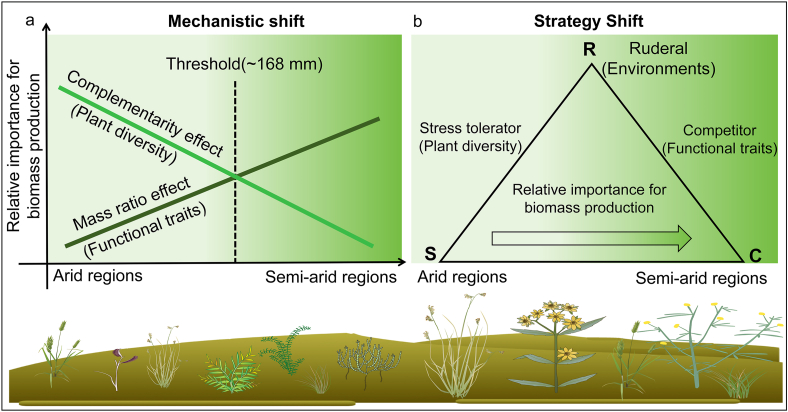
Schematic representation of the contribution of herbaceous plant diversity to community productivity. (a) The relationship between plant diversity and biomass production shifts from a complementarity effect in arid regions to a mass ratio effect in semi-arid regions. The green line illustrates the complementarity effect on biomass production driven by plant diversity at the *α* and *β* scales, while the dark green line represents the mass ratio effect driven by functional traits (e.g., community weighted mean (CWM) traits). The vertical dashed line indicates the transition threshold (∼168 mm MAP) between these two effects. (b) The strategy shift in plant communities along the aridity gradient, with stress-tolerator (S) and ruderal (R) strategies dominating in arid regions, and competitor (C) and ruderal (R) strategies becoming more prominent in semi-arid regions. The arrows indicate the directional influence of each strategy on biomass production, with the relative importance of these strategies changing across the gradient. The plants shown at the bottom represent the vegetation transition from arid (left) to semi-arid (right) regions, reflecting the corresponding shifts in plant strategies.

## 2 Materials and methods

### 2.1 Study site description

The studied region is situated along an East–West transect extending approximately 2100 km and traversing arid and semi-arid regions of North China (Fig. 2). The study area includes six major deserts of China: the Gurbantunggut Desert, Batangilin Desert, Ulanbuh Desert, Kubuqi Desert, Maowusu Desert, and Tengger Desert. These deserts form an ecological gradient transitioning from desert to desert-grassland, and grassland. The dominant plant species are *Haloxylon ammodendron*, *Agriophyllum squarrosum*, and *Stipagrostis pennata* (Hu et al., 2021). Prevalent soil types include aeolian sandy soils, grey desert soils, and loess. These soils are predominantly coarse-textured, with a strongly sand-dominated particle-size distribution (Table S1). The climatic conditions are characterised by a distinct precipitation gradient, with mean annual precipitation (MAP) in the range of 65–443 mm and mean annual temperature (MAT) of 3.1–9.6 °C (Su et al., 2021). Study sites were classified by geographic location using the global Köppen–Geiger climate classification map (http://koeppen-geiger.vu-wien.ac.at/present.htm). The region features a continental climate with abundant sunshine, receiving approximately 2500–3000 sunshine hours per year (Luo et al., 2021). It also experiences significant seasonal temperature fluctuations. Average monthly minimum temperatures drop to −27.4 °C in winter, while maximum monthly temperatures reach 41.2 °C in summer (Yao et al., 2022).

**Fig. 2:**
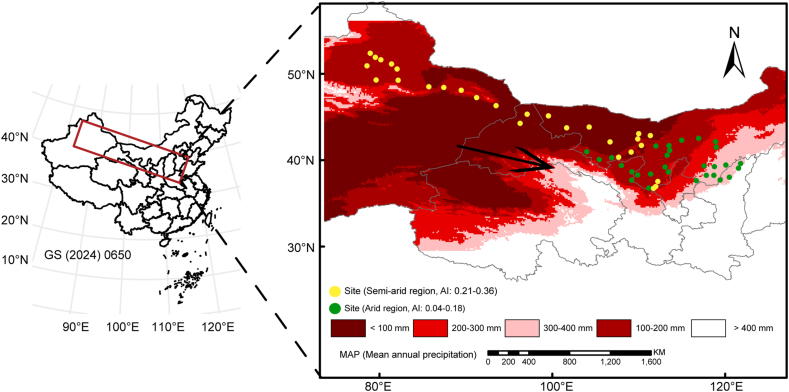
Study area and field sampling sites. Yellow and green points indicate arid region sites and semi-arid region sites.

### 2.2 Field investigation, plant and soil sampling, and property measurements

An extensive field survey was conducted in June and July 2021, coinciding with the peak of the plant growth season. Fifty-six survey sites were established along the precipitation gradient at 30–50-km intervals (27 in arid regions and 29 in semi-arid regions; Fig. 2). Well-vegetated, undisturbed lowland hills were selected to ensure consistent sampling conditions. We focused on herbaceous plants, which constitute over 70% of the species abundance and are crucial for nutrient cycling, ecosystem functioning, and adaptation to climate change (Zheng et al., 2022; Guo et al., 2023a). All herbaceous plants were treated as a single group; analyses focused on the total biomass and overall productivity of herbaceous communities, without separately considering the distinct life-history strategies of annual and perennial species. Each site (30 m × 30 m) comprised five equidistant 2 m × 2 m quadrats placed diagonally as biological replicates. Within each quadrat, all herbaceous species were catalogued. To ensure representativeness and minimise ontogenetic noise (Perez-Harguindeguy et al., 2013), five well-developed, mature individuals per species were selected for sampling. We measured plant height (H) and leaf length-width ratio (L/W). Additionally, seven fresh leaves were collected from various canopy orientations to calculate specific leaf area (SLA). Following field measurements, the total aboveground biomass (AGB) was harvested. Leaf samples were analysed for total carbon (TC) (Urbansky, 2001), total nitrogen (TN), and total phosphorus (TP) using a continuous flow analyser (Lambers, 2021). To account for the high heterogeneity of soil properties, five soil cores (10-cm deep) were collected from each subplot and combined into a single composite soil sample per subplot (i.e. five composite samples per site). The soil water content (SWC) was calculated based on the wet and dry weights of the soil samples (Zhang et al., 2023). Soil pH was measured using a pH meter (FiveEasy FE20, Switzerland) in a 1:2.5 soil/distilled water extract (Luo et al., 2021). Soil organic carbon (SOC) content was determined using the dichromate oxidation method (Urbansky, 2001). Soil total nitrogen (STN) content was measured using a continuous flow ion analyser (Shamrikova et al., 2022), and soil total phosphorus (STP) content was determined using the HClO_4_–H_2_SO_4_ ammonium molybdate-ascorbic acid method (Lambers, 2021).

### 2.3 Quantification of aboveground productivity

AGB was measured as the dry weight of plant material above ground, providing a direct indication of the current state of plant communities in dryland ecosystems (Davis et al., 2015; Taugourdeau et al., 2022; Zhang et al., 2023). Since most biomass is produced within a single growing season in arid and semi-arid environments, AGB serves as a reliable proxy for aboveground net primary productivity (Knapp and Smith, 2001; Huxman et al., 2004; Zuo et al., 2022).

### 2.4 Multiple attribute indicators of biodiversity

We used multidimensional diversity indices to assess biodiversity. Taxonomic (*α*) diversity, the most intuitive measure dependent on species richness, was expressed by counting the total number of species (equivalent to five biological replicates) across the five subplots at each site (Ulrich et al., 2014).

Functional (*α*) diversity was quantified using Rao’s quadratic entropy (RaoQ), which robustly integrates both the relative abundance of species and their pairwise functional differences (Morris et al., 2014; Botta-Dukát and Czúcz, 2016). We calculated functional diversity using species traits collected from each subplot. Six key traits were selected and classified into morphological traits (L/W, H, SLA) and chemical traits (Leaf C, N, P). To ensure mathematical comparability between these distinct trait categories (morphological vs chemical), all trait values were standardised prior to calculating the multidimensional functional distance matrix (Lepš et al., 2006; Villéger et al., 2008). These traits were selected because they are critical indicators of plant strategies to cope with water limitation and other environmental stresses associated with aridity gradients (Güsewell, 2004; Díaz et al., 2016). These traits also play pivotal roles in plant adaptation, survival, and competitiveness under varying moisture conditions (Wright et al., 2004; Reich, 2014). The ecological significance, inference scale, treatment level, and number of replicates of the six traits included in this study are detailed in Table S2.

For phylogenetic (*α*) diversity, the 123 plants surveyed were identified using the Plant List database (http://www.theplantlist.org/). The phylogenetic tree was constructed using the *V.PhyloMaker2* package in R (Jin and Qian, 2022). This method generates a phylogeny based on the GBOTB megatree backbone, which integrates molecular data from chloroplast (*rbcL*, *matK*) and nuclear (ITS) gene regions to ensure high topological resolution consistent with the APG IV system (Fig. S1). The Brownian motion evolutionary model was employed to test the phylogenetic conservatism of the six functional traits. Using Blomberg’s K statistic, observed trait values were compared with those predicted by the Brownian motion model (Table S3). The mean phylogenetic distance served as an indicator of phylogenetic (*α*) diversity, which characterises the average phylogenetic distance between species and reflects the absolute variation in community phylogenetic structure (Teichert et al., 2018).

Beta (*β*) diversity was estimated using the mean pairwise distance of the subplots within each site to the centroid, ensuring a uniform and unbiased assessment. Calculations of taxonomic, phylogenetic, and functional (*β*) diversity were all based on pairwise site dissimilarity methods. Taxonomic (*β*) diversity of herbaceous plant communities was assessed using the Bray–Curtis index based on species abundance at each site. Phylogenetic (*β*) diversity was determined using the phylogenetic tree of all sites and the calculated phylogenetic distance between species pairs. Functional (*β*) diversity was quantified based on the Euclidean distance of the six plant functional traits (Ulrich et al., 2014; Teichert et al., 2018).

The CWM measures the average state of species traits within a community, weighting each species by its proportion. The calculation of CWM incorporated leaf C, N, P content, L/W, SLA, and H. This approach ensures that the traits of dominant species, which contribute most to overall biomass and resource acquisition, are given more weight, thereby highlighting their role in driving community productivity and responses to environmental stress (Miller et al., 2019).

### 2.5 Quantification of complementarity effects, mass ratio effects and CSR strategies

To disentangle the ecological mechanisms driving community productivity and to visualise shifts in assembly strategies along the aridity gradient, we integrated the mass ratio and complementarity hypotheses within the theoretical framework of CSR strategies (Grime, 1977). We established a conceptual linkage between these ecological drivers and the three primary CSR strategies. We then quantified these strategies based on their relative explanatory power for AGB (Table 1).

**Table 1:** Summary of strategies, indicators, and ecological implications.

Strategy	Indicator	Ecological Implication
C	CWM index	Dominant species traits drive productivity (mass ratio effect)
S	Diversity indices	Coexistence of diverse strategies among subordinates enhances adaptation (complementarity effect)
R	Environmental factors	Spatial variability influences productivity

Note: “C” Competition strategy, “S” Stress tolerance strategy, and “R” Ruderal strategy.

We conceptualised the competition strategy (C-selection) as driven by the mass ratio effect. This posits that ecosystem functioning is determined by the traits of dominant species (Pierce et al., 2017). Consequently, we evaluated the CWMs of key functional traits to identify the primary trait-based drivers of competitive dominance.

We conceptualised the stress tolerance strategy (S-selection) as underpinned by the complementarity effect. This approach is based on the notion that higher multidimensional diversity (taxonomic, functional, or phylogenetic) enhances resource-use efficiency through niche differentiation, thereby facilitating survival and productivity under abiotic stress (Rosado and Mattos, 2017). Thus, we evaluated various diversity indices to represent this mechanism.

We conceptualised the ruderal strategy (R-selection) as being governed by environmental filtering. In dryland ecosystems, productivity is often constrained by stochastic environmental variability (Rosenfield et al., 2019). Therefore, we evaluated critical environmental limiting factors (e.g., precipitation dynamics and soil properties) to represent the external constraints driving opportunistic responses.

To quantify the realised CSR strategies, we employed a data-driven screening procedure. Based on linear mixed models (LMMs), the specific indicators with the strongest significant standardised effect sizes (i.e., highest explanatory power) within each category were selected as the final proxies for C, S, and R. The standardised regression coefficients (*β*) of these selected indicators were extracted and normalised to calculate the relative ternary coordinates (*Score*__*C, S, R*_) for each community:

$${Score}_{i}=\frac{\left|\beta_{i}\right|}{\left|\beta_{C}|+\right|\beta_{S}|+\left|\beta_{R}\right|}\times 100\%$$

where *i* represents the *C*, *S*, or *R* component. This methodological framework enables a quantitative, robust visualisation of the trade-offs among competition, niche complementarity, and environmental control (Fig. S2).

### 2.6 Abiotic variables

MAP and MAT data were acquired from the WorldClim database (www.worldclim.org) for each sampling site. These data have a spatial resolution of 30 arc-seconds (Shen et al., 2023). Additionally, we calculated the aridity index (AI) for each site, defined as the ratio of annual precipitation to annual potential evapotranspiration, sourced from the Global Aridity Index and Potential Evapotranspiration Climate Database (https://cgiarcsi.community/) (Hu et al., 2021). We also recorded the longitude, latitude, and altitude of each sampling site. These variables were then used to interpret the environmental conditions and ecosystem (Pointing and Belnap, 2012).

### 2.7 Statistical analysis

To assess the variability of AGB, plant diversity, and functional traits across different climatic regions (arid and semi-arid regions), we first calculated the coefficient of variation (*CV*) using the original data. The *CV* is defined as the standard deviation (*σ*) divided by the mean (*μ*). Subsequently, normality tests and *Z*-score standardisation were applied to improve the normality and homogeneity of residual variances for further analysis. A three-level nested analysis of variance (ANOVA) (between sites, between plots within sites, and within plots) (Xie et al., 2022) was performed to test the spatial distribution of these indicators and assess the sources of variability at different levels.

To explore the relationship between plant diversity, functional traits, and environmental variables with AGB, LMMs were constructed using the *lme4* package (site as a random effect and REML for variance component estimation; Eqs. A1-1, A2-1, and A3-1 in Table S4). The variance inflation factor (*VIF*) was used to assess collinearity among predictors, and any variable with a *VIF* > 10 was excluded. Plant diversity and functional trait indices of all sites had *VIFs* below 10; the environmental variables, including longitude, elevation, SWC, and AI, were removed (Table S5). The models also employed the Akaike information criterion (*AIC*), and Δ*AIC* < 2 was used to identify the best predictors of AGB (Le Bagousse-Pinguet et al., 2019). The final models (Table S4; Eqs. A1-2, A2-2, and A3-2) were used to calculate the relative weights of each predictive variable based on their R^2^ values to examine the contributions of complementarity and mass ratio effects.

To assess the relative importance of biotic and abiotic drivers on AGB, we constructed a piecewise structural equation model (SEM) using the piecewiseSEM package. The modelling process began with specification of an initial *a priori* model (Fig. S3). This model represented our hypothesised causal network linking climate and soil conditions to community functional structure (traits and diversity) and ecosystem function. This global network was operationalised by decomposing it into a set of local LMMs. We optimised the model structure by stepwise removal of non-significant pathways to enhance parsimony. Global model fit was evaluated using Fisher’s C statistic; *p* > 0.05 indicated no significant deviation between the hypothesised causal structure and the observed data. Furthermore, *AIC* was used to assess model robustness, with lower *AIC* values indicating superior predictive accuracy. Within this analytical framework, we explicitly tested two competing ecological hypotheses: significant direct effects of multidimensional diversity on AGB were interpreted as support for the complementarity effect, whereas strong direct regulation by dominant trait values (CWM) was taken as evidence for the mass ratio effect.

To detect potential thresholds where the relationship between plant diversity (or functional traits) and AGB changes along a critical environmental gradient, we applied a rolling-window modelling framework across the full study extent. Sampling units were ordered along the focal environmental gradient, and overlapping windows spanning k observations were generated to ensure continuous coverage of the gradient. Within each window, we applied Eq. A3-2 and extracted the standardised fixed-effect coefficients to facilitate comparison across predictors. The coefficients were then bootstrapped (500 iterations per window) to obtain the median estimate and its 95% confidence interval for each predictor. These window-level coefficient summaries were subsequently regressed against the environmental gradient to characterise how effect sizes varied along the gradient and to identify candidate breakpoints. Potential thresholds were quantified using hinge (piecewise) regression on the coefficient–gradient relationships, and breakpoint uncertainty was summarised using *AIC* profile–based 95% confidence intervals. To evaluate whether a candidate breakpoint represented a significant “shift”, we compared a single-slope model against a segmented model using *ΔAIC* and likelihood ratio/ANOVA tests. A window size of k = 10 was selected as it provided the optimal balance between local estimation stability (requiring sufficient data per window) and resolution along the gradient to detect potential non-linearities, as confirmed by sensitivity analysis (Table S6). After identifying the precipitation threshold, we verified these ecological shifts by applying discontinuous piecewise linear regression to the raw data. This approach facilitated the detection of potential regime shifts by permitting intercept discontinuities. Model validity and slope divergence were confirmed via *ΔAIC* and slope shift tests (*P*_*shift*_). All rolling-window construction, mixed-model fitting, and bootstrapping procedures used the functions ‘lmer’, ‘bootMer’, ‘makeCluster’, ‘registerDoSNOW’, and ‘runner’.

To operationalise the CSR strategy framework and quantify community-level adaptation strategies across climatic regions, we implemented a robust multi-step analytical procedure. First, we extracted the standardised regression coefficients (*β*) of the potential indicators from the optimal LMMs (Eq. A3-2). These coefficients served as proxies for the relative strength of each ecological driver. To mitigate multicollinearity and integrate multidimensional information, principal component analysis (PCA) was employed for any strategy category represented by multiple intercorrelated variables (e.g., multiple functional traits for C-selection). The first principal component (PC1) was retained as the composite indicator (see Text S1 for the detailed ecological and statistical rationale). Subsequently, the absolute standardised coefficients for the final proxies of C, S, and R were normalised to calculate their relative ternary coordinates. This ensured that the sum of the three strategy components equalled 100%. Finally, the realised CSR strategies were visualised using the ‘*ggtern*’ package. In this triangular topological space, the proximity of data points to the vertices indicates a functional specialisation driven by a single dominant mechanism. Clustering in the central area reflects a generalist strategy in which multiple drivers exert balanced influence at intermediate levels of stress and disturbance. All data analyses and visualisations were performed in R (4.1.0).

## 3 Results

### 3.1 Variation in plant diversity, functional traits and aboveground biomass in different climatic regions

In arid regions, the *CV* for plant diversity, functional traits, and AGB indicators ranged from 7% to 21%, with FD(*α*), CWM.H, and CWM.SLA exhibiting particularly high variability (> 20%). In contrast, the variation of most indicators in the semi-arid regions was considerably lower (Fig. 3a). This reduced variability implies that stronger environmental filtering or biotic constraints in semi-arid regions may drive functional convergence, leading to more homogeneous communities. Nested ANOVA-based analyses showed that more than 50% of the variance across all indicators was due to differences between sample sites, highlighting the impact of large-scale environmental change on habitat heterogeneity (Fig. 3b).

**Fig. 3:**
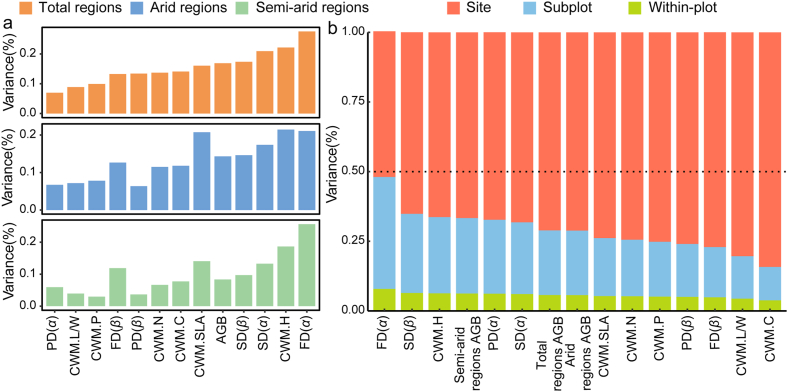
Characteristics and sources of variation in plant diversity, functional traits and aboveground biomass in different climatic regions. (a) Variation characteristics of plant diversity, functional traits, and aboveground biomass across different climatic regions; (b) sources of variation in plant diversity, functional traits, and aboveground biomass at the site, subplot, and within-plot levels. FD (*α*), functional alpha diversity; TD (*β*), taxonomic beta diversity; CWM.H, community weighted mean height; Semi-arid regions AGB, aboveground biomass in semi-arid regions; PD(*α*), phylogenetic alpha diversity; TD (*α*), taxonomic alpha diversity; Total region AGB, aboveground biomass in total regions; Arid regions AGB, aboveground biomass in arid regions; CWM.SLA, community weighted mean specific leaf area; CWM.N, community weighted mean nitrogen content; CWM.P, community weighted mean phosphorus content; PD (*β*), phylogenetic beta diversity; FD (*β*), functional beta diversity; CWM.L/W, community weighted mean leaf length-width ratio; CWM.C, community weighted mean carbon content. Site is the variance among the different sites, subplot is the variance among the subplots, and Within-plot is the variance within the subplots.

### 3.2 The relative importance of plant diversity and environmental factors on aboveground biomass in different climatic regions

LMMs indicated that plant diversity (FD (*α*)), functional traits (CWM.H and CWM.SLA), and environmental factors (latitude, MAP, and soil pH) significantly influenced AGB accumulation across the climatic regions (Fig. 4 and Table S7). Crucially, variance partitioning revealed a distinct mechanistic shift along the aridity gradient. In arid regions, FD (*α*) was the primary contributor to AGB variability. Consequently, the complementarity effect (33.85%) was the dominant driver, explicitly outweighing the mass ratio effect (27.52%). In contrast, a notable reversal occurred in semi-arid regions. The contribution of dominant traits (CWM.H and CWM.SLA) increased substantially; the mass ratio effect (37.97%) surpassed the complementarity effect (30.23%). Across the study region, the mass ratio effect (39.88%) generally prevailed over the complementarity effect (29.47%). This dominance was driven largely by the substantial contribution of CWM.H (24.09%) (Fig. 5). This pattern highlights the scale-dependence of these assembly mechanisms.

**Fig. 4:**
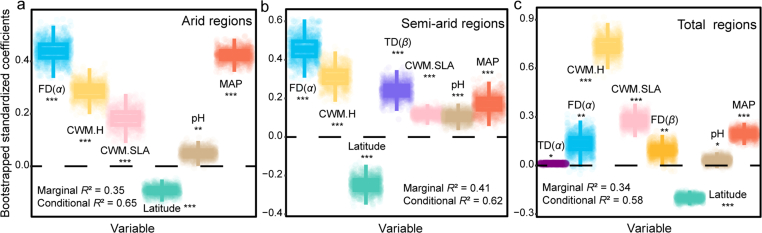
Standardised regression coefficients and 95% confidence intervals for the linear mixed model predictor variables. The dependent variable is aboveground biomass (AGB). The standardised regression coefficients for each predictor variable are shown in Table S5. Significant codes: 0, “∗∗∗” 0.001, “∗∗” 0.01, “∗” 0.05. FD (*α*), functional alpha diversity; TD (*β*), taxonomic beta diversity; CWM.H, community weighted mean height; TD (*α*), taxonomic alpha diversity; CWM.SLA, community weighted mean specific leaf area; FD (*β*), functional beta diversity; MAP, mean annual precipitation.

**Fig. 5:**
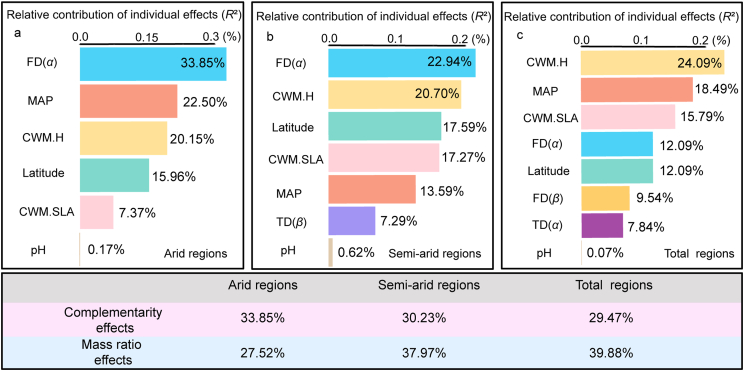
Contributions of linear mixed models to predict variables. In arid regions, the complementarity effect is represented by the effect of FD (*α*), while the mass ratio effect is represented by the combined effects of CWM.H and CWM.SLA. In semi-arid regions, the complementarity effect is represented by the combined effects of FD (*α*) and TD (*β*), while the mass ratio effect is represented by the combined effects of CWM.H and CWM.SLA. In the total regions, the complementarity effect is represented by the combined effects of FD (*α*), FD (*β*), and TD (*α*), while the mass ratio effect is represented by the combined effects of CWM.H and CWM.SLA. FD (*α*), functional alpha diversity; TD (*β*), taxonomic beta diversity; CWM.H, community weighted mean height; TD (*α*), taxonomic alpha diversity; CWM.SLA, community weighted mean specific leaf area; FD (*β*), functional beta diversity; MAP, mean annual precipitation.

**Fig. 6:**
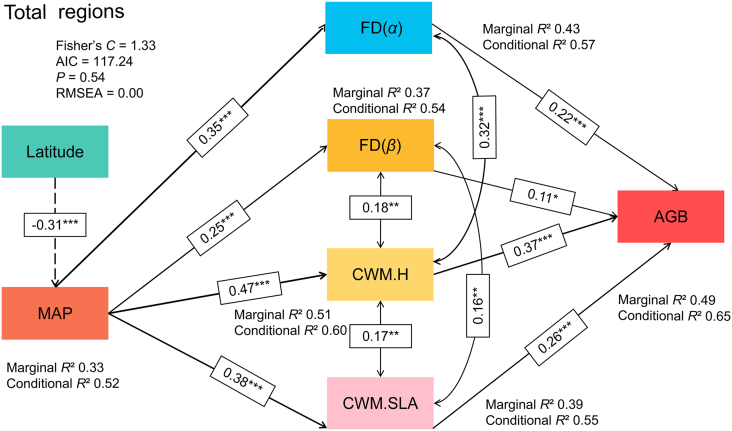
Direct and indirect effects of abiotic and biotic variables on plant aboveground biomass in the whole study region. FD (*α*), functional alpha diversity; CWM.H, community weighted mean height; CWM.SLA, community weighted mean specific leaf area; FD (*β*), functional beta diversity; MAP, mean annual precipitation; AGB, aboveground biomass. Double-headed arrows indicate covariance trends.

In arid regions, SEM analyses showed that FD (*α*) and CWM values had a positive direct effect on AGB. The direct effect of FD (*α*) on AGB (0.52) was significantly higher than that of CWM.H (0.30) and CWM.SLA (0.15). MAP significantly contributed to changes in FD (*α*) (0.54), CWM.H (0.41), and CWM.SLA (0.34). Notably, CWM values showed a significant positive effect on AGB, while CWM.H and CWM.SLA showed negative synergistic effects (−0.25; statistically significant, indicating covariance trends) (Fig. S4). In semi-arid regions, FD (*α*) and CWM.H showed almost the same positive direct effect on AGB as in arid regions, which were 0.56 and 0.50, respectively; they also exhibited high positive synergy (0.49, statistically representing covariance trends) (Fig. S5). For the whole study region, SEM explained 49% of the variation in AGB. MAP remained the critical environmental factor, significantly influencing the variation in FD and CWM. The positive direct effect of CWM.H (0.37) and CWM.SLA (0.26) on AGB increased significantly, and the relationship between them also shifted to positive synergy (0.17; statistically representing covariance trends) (Fig. 6).

### 3.3 The thresholds of critical environmental factors driving the relationship between plant diversity and aboveground biomass production in the whole region

**Fig. 7:**
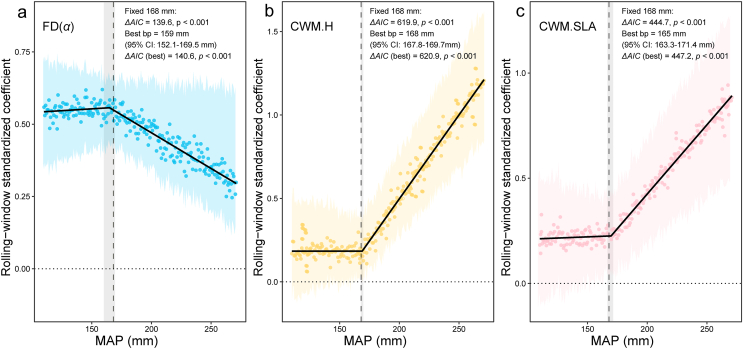
Rolling-window standardised coefficients of community biomass (AGB) against mean annual precipitation (MAP) and breakpoint detection. For each sliding window (window size *k* = 10 plots; bootstrap B = 500), we fitted a multiple linear model of standardised AGB as a function of standardised predictors (FD (*α*), CWM.H, and CWM.SLA). Points represent the bootstrap median of the standardised regression coefficient (*β*) estimated for each window, plotted at the mean MAP of the window. Shaded ribbons indicate the 95% bootstrap confidence interval (95% CI: 2.5–97.5%) of the coefficient for each window. The horizontal dotted line denotes *β* = 0. The vertical dashed line marks the *a priori* threshold at MAP = 168 mm, and the light grey band indicates the 95% confidence interval of the statistically estimated breakpoint derived from an *AIC*-profile hinge (piecewise linear) regression fitted to the rolling-window coefficient series (*β* vs. MAP). Importantly, to prevent potential misinterpretation, we note that for the breakpoint calculation, this hinge regression was fitted directly to the rolling-window coefficients (i.e., the points shown in the plot), rather than to the raw data. Solid black lines show the best-fitting hinge model (single-breakpoint segmented regression) for each predictor panel. The MAP axis shows the range of window-median MAP values for which rolling-window coefficients could be estimated (window size *k* = 10), rather than the full MAP range of all plots; consequently, the MAP range is narrower than the raw climatic gradient. FD (*α*), functional alpha diversity; CWM.H, community weighted mean height; CWM.SLA, community weighted mean specific leaf area; MAP, mean annual precipitation.

**Fig. 8:**
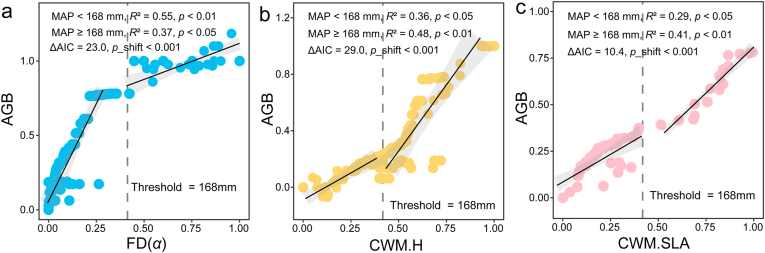
Validation of mean annual precipitation (MAP) as the threshold for plant diversity, functional traits, and aboveground biomass. Relationships between aboveground biomass (AGB) and each predictor (a, FD (*α*); b, CWM.H; c, CWM.SLA) are shown separately for plots with MAP < 168 mm and MAP ≥ 168 mm. Solid lines show ordinary least squares fits within each MAP group; grey ribbons denote the 95% confidence band of the fitted regression within each group. The vertical dashed line indicates the MAP threshold (168 mm). To formally test whether the AGB–trait relationship exhibits a slope shift at 168 mm, we compared (i) a single-slope linear model, AGB ∼ FD (*α*)/CWM.H/CWM.SLA, against (ii) an interaction model allowing different slopes between MAP groups, AGB ∼ FD (*α*)/CWM.H/CWM.SLA × MAP_grp, where MAP_grp is defined by the 168 mm threshold. Notably, this slope-shift test was performed directly on the raw AGB-trait data. The reported *ΔAIC* equals *AIC* (single-slope model) – *AIC* (interaction model), so positive values indicate improved fit when allowing group-specific slopes. The reported *p* value (*p*_sh_ᵢ_ft_) is the significance of the interaction term (equivalently, the nested-model comparison), quantifying whether slopes differ between MAP groups. FD (*α*), functional alpha diversity; CWM.H, community weighted mean height; CWM.SLA, community weighted mean specific leaf area; MAP, mean annual precipitation.

MAP was the dominant gradient modulating biodiversity– and trait–productivity relationships across the study region. Rolling-window coefficients indicated a pronounced transition around ∼168 mm MAP. FD (*α*) showed a positive effect on AGB at low MAP, but this effect declined with increasing precipitation. The best-supported breakpoint occurred at 159 mm (95% CI: 152.1–169.5; Fig. 7a). In contrast, the effects of dominant-trait metrics strengthened with MAP, with best-supported breakpoints at 168 mm for CWM.H (95% CI: 167.8–169.7; Fig. 7b) and 165 mm for CWM.SLA (95% CI: 163.3–171.4; Fig. 7c). Fixing the breakpoint at 168 mm yielded a substantially improved model fit relative to a single-slope alternative (*ΔAIC* = 139.6 for FD(*α*), 619.9 for CWM.H, and 444.7 for CWM.SLA; *p* < 0.001; Fig. 7). Consistently, piecewise relationships showed stronger diversity control of AGB below 168 mm, whereas trait control was stronger above 168 mm (Fig. 8).

### 3.4 CSR framework model on aboveground biomass production in different climatic regions

**Fig. 9:**
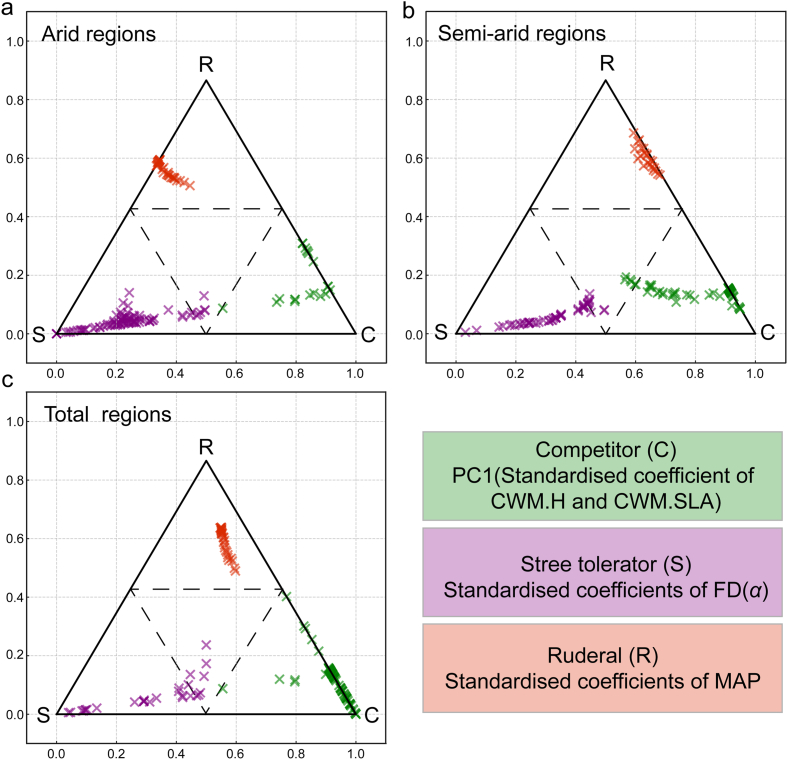
CSR triangulation of plant diversity, functional traits and environmental variables on aboveground biomass. The ternary plots display the functional trajectory of plant communities in (a) Arid regions, (b) Semi-arid regions, and (c) Total regions. Each data point represents an individual sampling subplot (*n* = 135 for arid; *n* = 145 for semi-arid). The coordinates were calculated by normalizing the standardised regression coefficients (*β*) derived from the LMMs, representing three distinct assembly mechanisms: Competitor (C): associated with the mass ratio effect, quantified by the standardised coefficients of CWM traits (*β*_*PC1(CWM)*_). Stress tolerator (S): associated with the complementarity effect, quantified by the standardised coefficients of functional diversity (*β*_*FD(α)*_). Ruderal (R): associated with environmental Filtering, quantified by the standardised coefficients of precipitation (*β*_*MAP*_), reflecting opportunistic responses to resource pulses. Statistics: in arid regions (a), communities were primarily driven by S-selection (81 subplots) and R-selection (29 subplots). In semi-arid regions (b), communities shifted towards C-selection (66 subplots) and S-selection (52 subplots). Points falling in the central triangle represent communities with a generalist strategy, regulated by a trade-off among multiple mechanisms. In this triangular space, proximity to the vertices indicates functional specialization driven by a single dominant mechanism. Conversely, clustering in the central area reflects a generalist strategy. This central zone represents communities where productivity is governed by a balanced combination of competitive dominance, niche complementarity, and environmental opportunism under intermediate stress and disturbance. FD (*α*), functional alpha diversity; CWM.H, community weighted mean height; CWM.SLA, community weighted mean specific leaf area; MAP, mean annual precipitation.

Utilising the CSR theoretical framework, we developed an interaction model to assess AGB variability. This model incorporated the standardised regression coefficients of key biotic indicators (FD (*α*), CWM.H, and CWM.SLA) and a single environmental indicator, MAP. The S and R strategies were predominant in arid regions (Fig. 9). The blend of C and R strategies in the semi-arid regions highlighted the dual significance of stress resistance and competitive resource utilisation in AGB accumulation. Across the study region, the prominence of the C strategy underscored the essential contribution of competitive traits in AGB across various environmental gradients (Fig. 9).

## 4 Discussion

### 4.1 Contribution of complementarity and mass ratio effects to community aboveground productivity

Consistent with our first hypothesis, the results indicated that community productivity in arid and semi-arid regions was predominantly influenced by complementarity (FD (*α*); 33.85%) and mass ratio effects (CWM.H and CWM.SLA; 37.97%). The relative importance of complementarity is expected to increase in arid environments because severe water limitation strengthens both niche differentiation and stress-ameliorating positive interactions among neighbours (Bertness and Callaway, 1994; Soliveres et al., 2015). In resource-limited arid regions, distinct species can exploit resources at different spatial or physiological levels, which reduces competition and increases resource utilisation (Craven et al., 2018; Delgado-Baquerizo et al., 2020). Crucially, beyond niche partitioning, facilitation can further amplify diversity–productivity linkages in drylands. For example, canopy-forming or tussock-forming species may buffer microclimatic extremes (lowering soil temperature and evaporative demand) to improve the establishment and performance of neighbouring herbs (Butterfield et al., 2013; Soliveres et al., 2015). In addition, deep-rooted species can redistribute soil water to shallow layers via hydraulic lift during dry periods, indirectly benefiting co-occurring shallow-rooted species (Prieto et al., 2012). Therefore, a greater contribution of FD (*α*) enhances not only the diversity of resource-use strategies but also the structural heterogeneity required for these positive interactions (Oliveira et al., 2016). Our FD (*α*) index (Rao’s Q) effectively captures this multidimensional variation in traits (e.g. differences in canopy architecture and rooting depth), which directly reflect the facilitation potential among co-occurring species under severe drought stress. Similarly, predominance of the complementarity effect was reported in the Gurbantunggut Desert (Guo et al., 2023b). Herbaceous plant communities with higher diversity may be more resilient to disturbances such as drought because alternative species can occupy vacant ecological niches (Fargione et al., 2007). Moreover, the reduced contribution of the mass ratio effect could stem from convergent evolution in arid environments, where species evolve similar functional traits to cope with abiotic stress. Therefore, complementarity effects, encompassing both niche differentiation and facilitation, are critical for sustaining community productivity in arid regions (Prado-Junior et al., 2016; Williams et al., 2017).

Although we found a lower contribution of the mass ratio effect to AGB in arid regions, it nevertheless dominated in semi-arid regions and across the study area. This shift can be explained by the increased prevalence and influence of dominant species in semi-arid regions owing to relatively more favourable environmental conditions so that the functional traits of dominant species exert a stronger influence on community productivity. Consequently, when considering contributions from both arid and semi-arid regions collectively, the mass ratio effect emerged as the dominant mechanism driving productivity at the regional scale (LaManna et al., 2017). For example, species with greater plant height or specific leaf area might have competitive advantages in light acquisition and nutrient utilisation strategies, advantages directly related to the productivity of individual plants (Koerner et al., 2018). Therefore, some dominant species may completely occupy a specific ecological niche, suppressing the productivity of other species within the same niche (Brandt et al., 2017; Shen et al., 2023), thereby strengthening the mass ratio effect. It Plant height and specific leaf area are also considered key selective traits for high-productivity environments in dryland ecosystems (Luo et al., 2023). Hence, decreased aridity may increase community productivity by selectively promoting these key traits (Peñuelas et al., 2017; Grünzweig et al., 2022). Simultaneously, dominant species tend to recur and, through competition, determine the upper limits of community function and productivity. For example, *Stipa breviflora* and *Corispermum heptapotamicum*, common species in these environments, have traits such as deep root systems and high SLA, allowing them to outcompete other species for resources and significantly influence community productivity (Paquette and Messier, 2011; La Pierre et al., 2016; Cavender-Bares et al., 2022). Therefore, the key functional characteristics of dominant species are critical to achieve more efficient ecological management and establish better conservation strategies in dryland ecosystems. Additionally, the complex role of diversity in maintaining community productivity at local and regional scales must be recognised.

### 4.2 The dynamic relationship between plant diversity and aboveground productivity under environmental thresholds

SEM confirmed that both complementarity and mass ratio effects contribute significantly to herbaceous plant community productivity. The relationship between CWM.H and CWM.SLA with respect to biomass production exhibited pronounced scale-dependent differentiation. The negative synergy (−0.25) in arid localities, highlights a fundamental hydraulic trade-off. Taller plants, subject to higher xylem tension, are more susceptible to hydraulic failure and carbon starvation under a water deficit (Isbell et al., 2019; Adeleye et al., 2023). Consequently, to ensure survival, they adopt conservative strategies—investing in high-density tissues rather than expansive leaf areas (McDowell et al., 2008). This height–safety trade-off restricts the simultaneous maximisation of vertical growth and photosynthetic efficiency in resource-poor environments (Maestre et al., 2021; Medeiros et al., 2023). Across the study region, these traits shift to a positive synergy driven by the relaxation of environmental filtering (0.17). This pattern aligns with findings from broader Chinese grassland transects, where precipitation and soil nutrients drive the coordinated increase of plant size and diversity (Ma et al., 2010). As resource availability improves, the constraints of the ‘survival–growth’ trade-off diminish, allowing communities to adopt a ‘bigger and faster’ strategy (Balazs et al., 2022; Yan et al., 2023). Furthermore, we found positive synergy (0.16) between FD (*β*) and CWM.SLA, suggesting that productive communities maintain higher spatial heterogeneity. In high-SLA regions, functional turnover is likely driven by biotic competition and micro-environmental partitioning. This mirrors dynamics observed in Mediterranean ecosystems, where soil nutrient heterogeneity modulates functional group interactions, thereby preventing biotic homogenisation even under semi-arid conditions (García-Palacios et al., 2011). Thus, while arid stress imposes convergence (low FD (*β*)), resource-enriched patches promote divergence, enhancing the ecosystem’s capacity to buffer environmental fluctuations (Huang et al., 2018; Yuan et al., 2020; Wang et al., 2022a, b).

Environmental factors, particularly MAP, can modulate the relationship between plant diversity and productivity, indicating the presence of environmental thresholds. Across the study region, an inflexion points in the relationship between FD (*α*), CWM.H, CWM.SLA, and productivity was identified at a MAP value of approximately 168 mm. Below this threshold, functional complementarity represented by FD (α), such as deeper root systems and smaller or thicker leaves, likely reduces direct competition for resources. This reduction allows the community to utilise available resources more efficiently and support productivity gains. Additionally, facilitative interactions may play a significant role in these environments. Notably, although these mechanisms are considered contributing factors, the exact mechanisms that currently improve productivity cannot be determined. This dynamic indicates a fundamental shift in the mechanisms governing productivity due to varying moisture conditions (Zhang et al., 2017; Ricotta et al., 2023). In contrast, when MAP exceeds 168 mm, moisture ceases to be the primary limiting factor for plant growth in semi-arid regions and instead productivity increasingly relies on morphological and physiological traits of plant species. In this context, functional traits such as CWM.H and CWM.SLA become key indicators of plant competitiveness and leaf construction costs. Changes in these traits reflect plants’ tendency to enhance resource acquisition efficiency and productivity by increasing their height and specific leaf area. Overall, as precipitation surpasses this critical threshold, the composition of plant communities gradually shifts from species adapted to arid environments to those characterised by greater height and specific leaf area, traits more suited to relatively water-abundant conditions. This shift suggests an adaptive succession of plants in response to changing environmental moisture conditions (Zhang et al., 2012; Coe and Sparks, 2014; Ruppert et al., 2015).

Although the network of 56 sites (27 arid and 29 semi-arid) ensures robust spatial representativeness across the transition zone, the potential influence of the moderate sample size on the precise stability of the statistical models must be acknowledged. Critically, this identified threshold (168 mm) is notably lower than the conventional ecological boundary (typically 200–250 mm). To verify that this threshold represents a genuine ecological transition rather than a statistical artefact, we further examined shifts in community composition along the gradient. Our analysis confirmed a distinct structural change in species dominance and community composition occurring specifically around the 168 mm mark (Fig. S6). The deviation of this biological threshold from the conventional climatic boundary (typically 200–250 mm) may be attributed to three specific factors. First, this study focused exclusively on herbaceous communities, which are likely more sensitive to water fluctuations than whole-ecosystem assessments that include deep-rooted woody vegetation. Second, the widely distributed sandy soils in the study area (average sand content > 94%) have a field capacity significantly lower than that of loamy soils. Consequently, the actual plant-available water is much lower than reflected by MAP values. Therefore, the shift of the ecological threshold towards a lower MAP might be a combined result of the physical properties of sandy soils and climatic factors (Table S1). Third, use of gridded WorldClim data (∼1 km resolution) might slightly underestimate local micro-topographic variations and effective precipitation during the growing season. Although the precise numerical threshold may be specific to our herbaceous communities and edaphic context, our study demonstrates that such ecological mechanisms can shift at precipitation levels distinct from broad climatic classification boundaries. Consequently, this threshold represents a contingent boundary specific to the herbaceous layer and local edaphic context rather than a generalised geographic limit.

### 4.3 Trade-offs in ecological strategies for aboveground productivity of herbaceous plant communities and their adaptive management strategies

The CSR theory findings further supported our second hypothesis (Fig. 9). In arid regions, the predominance of the S strategy in herbaceous plants suggested that these plants can withstand extreme environmental pressures such as prolonged droughts, manifested in traits such as deeper roots and smaller or water-retaining leaves. These features enable the plants to survive in water-scarce environments and rapidly utilise resources under favourable conditions (Munoz et al., 2016; Gallagher et al., 2020). Therefore, the importance of these specific traits warrants further investigation. The dominance of the S strategy does not necessarily imply trait convergence. Rather, strong stress filtering can select for multiple, non-redundant drought-survival syndromes (e.g. contrasting rooting depth, phenology, and leaf economic traits). This selection process ultimately increases interspecific trait differentiation and elevates functional diversity to strengthen complementarity by partitioning limiting resources across space and time while reducing competitive overlap under chronic water limitation (García-Palacios et al., 2011). The partial contribution of the R strategy indicates that some plants in these communities can grow and reproduce swiftly during periods of intermittent resource abundance (e.g. water availability following rainfall events). By exploiting short-lived resource pulses, R-strategy species may further broaden temporal niche breadth and reinforce complementarity in arid communities, thereby contributing to community productivity (Pierce et al., 2017).

A combination of C and S strategies emerged as crucial in semi-arid regions. Outside of intermittent drought periods, plants compete for limited resources besides water. For example, they may expand their leaf areas or accelerate growth to occupy more space (Caccianiga et al., 2006; Hodgson et al., 2011). In these environments, plants may need to balance survival and competitive strategies to optimise resource utilisation and productivity (Jabot and Pottier, 2012; Mason-Jones et al., 2022). As moisture limitation relaxes, C strategists can increasingly dominate community biomass through acquisitive traits such as tall stature and high SLA, conferring disproportionate advantages in light capture and carbon gain. This provides a direct mechanistic link to the mass ratio effect: ecosystem functioning becomes progressively governed by the trait values of biomass-dominant species, as captured by shifts in CWM.H and CWM.SLA (Ricotta et al., 2023). The dominance of the C strategy across the study region highlighted that competitive traits such as larger leaf areas, greater plant height, and higher growth rates are crucial for promoting productivity over a wider environmental gradient (Rosado and Mattos, 2017). These traits enable plants to maximise growth under favourable conditions. This capacity allows them to accumulate more biomass, providing a distinct advantage under shifting environmental regimes (Bruelheide et al., 2018). Overall, the predominance of different strategies in various environmental settings reflects the adaptability and varied responses of plant communities to distinct environmental stresses (Wilson et al., 1999; Jabot and Pottier, 2012; Firn et al., 2019). A preference for S and R strategies may arise from the adaptation to long-term drought in arid regions. A blend of S and C strategies in semi-arid regions may reflect adaptation to intermittent droughts and resource fluctuations. Therefore, understanding these dynamic interactions is vital for maintaining productivity and ensuring sustainable management and restoration in dryland ecosystems.

Adaptive management strategies for dryland plants aimed at maximising survival and productivity under extreme drought conditions rely on physiological, morphological, and ecological adaptations (Ruppert et al., 2015). In regions with MAP < 168 mm, where productivity is primarily supported by the complementarity effect, restoration and conservation should prioritise maintaining and rebuilding high functional diversity in herbaceous communities. This entails introducing mixtures of functionally dissimilar, drought-adapted herbaceous species that span contrasting water-acquisition and persistence strategies (e.g. deep-vs shallow-rooted species, conservative vs acquisitive leaf traits). This approach enhances community-level resource partitioning and buffers against rainfall intermittency (Schöb et al., 2013; Wehn et al., 2017; Bricca et al., 2021; Zheng et al., 2022). Additionally, monoculture or low-diversity seed mixes should be avoided, as they are less likely to deliver the complementarity benefits observed in our arid sites. Protection efforts should focus on remnant herbaceous communities that already exhibit high FD, as these represent key reservoirs of drought-resilient functional strategies. Monitoring schemes should therefore track functional composition (trait distributions/FD) alongside species richness, rather than relying solely on taxonomic metrics (Norris et al., 2011; Eskelinen et al., 2012; Granda et al., 2018). In regions with MAP > 168 mm, where the mass ratio effect becomes more influential, management should emphasise screening and promoting native dominant species (or species combinations) with trait syndromes associated with higher productivity. Particularly, greater CWM.H and higher CWM.SLA should be targeted. For example, in our semi-arid sites, robust species such as *Peganum nigellastrum* and *Corispermum heptapotamicum* exhibited high CWM.H and CWM.SLA, offering strong candidates for restoration seeding. This can be operationalised by using local provenance seed sources and selecting candidate dominants that reliably attain taller stature and higher SLA under local conditions, while maintaining sufficient establishment success and persistence (Laughlin and Moore, 2009; Parolari et al., 2015). Restoration designs in this region can therefore prioritise the identity and performance of a small set of productive dominants, complemented by subordinate species for stability and resistance, rather than maximising diversity per se (Díaz and Cabido, 2001; Valencia et al., 2015). Management actions such as density optimisation and periodic adjustment (e.g. selective thinning or gap-filling reseeding) can help sustain a dominance structure and maintain productivity over time (Chitale et al., 2012; Ruppert et al., 2015; Felton et al., 2021). These targeted recommendations provide a more direct basis for biodiversity-informed dryland restoration under increasing climatic variability.

## 5 Conclusions

This study provides empirical evidence supporting a critical role of biodiversity in buffering productivity fluctuations in natural dryland ecosystems. By revealing the transition from complementarity effects in arid regions to mass ratio effects in semi-arid regions, this study provides valuable insights into how plant diversity and functional traits influence ecosystem productivity across environmental gradients. Furthermore, the identified MAP threshold of 168 mm serves as a critical demarcation between arid and semi-arid regions, highlighting the role of plant strategies in shaping ecosystem productivity. Below this threshold, promoting functional diversity may improve resource-use efficiency, whereas focusing on traits such as plant height and specific leaf area could maximise biomass production above the threshold. Ultimately, our findings demonstrate that the functional role of biodiversity in drylands is not static but shifts predictably along moisture gradients. Effective ecosystem management must therefore be context-dependent, targeting functional diversity in arid zones and dominant-trait optimisation in semi-arid zones, as guided by ecological thresholds.

## Data availability statement

The data that support the findings of this study are openly available in the Science Data Bank at https://www.scidb.cn/anonymous/WkZibWUy.

## CRediT authorship contribution statement

X.-B.Z.: Conceptualization, Methodology, Writing - Review & Editing. Y.-M.Z.: Conceptualization, Methodology, Writing - Review & Editing. H.G.: Investigation, Formal analysis, Writing - Original Draft. J.-F.Y.: Investigation, Formal analysis. B.-F.Y.: Investigation. H.-H.M.: Writing - Review & Editing. T.L.: Writing - Review & Editing.

## Declaration of competing interest

The authors declare that they have no known competing financial interests or personal relationships that could have appeared to influence the work reported in this paper.

## Figures and Tables

**figs1:**
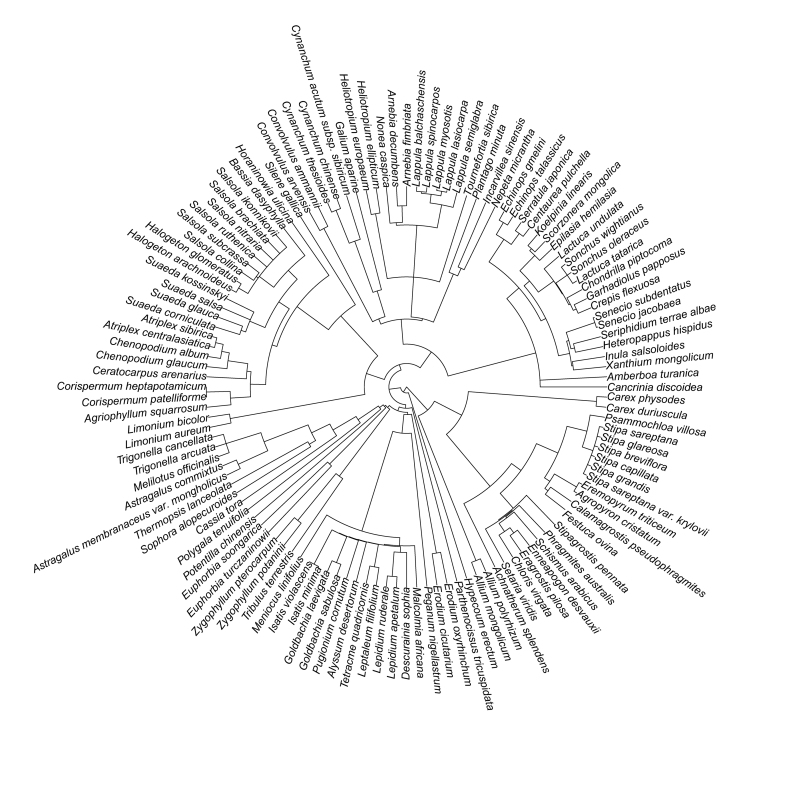


**figs2:**
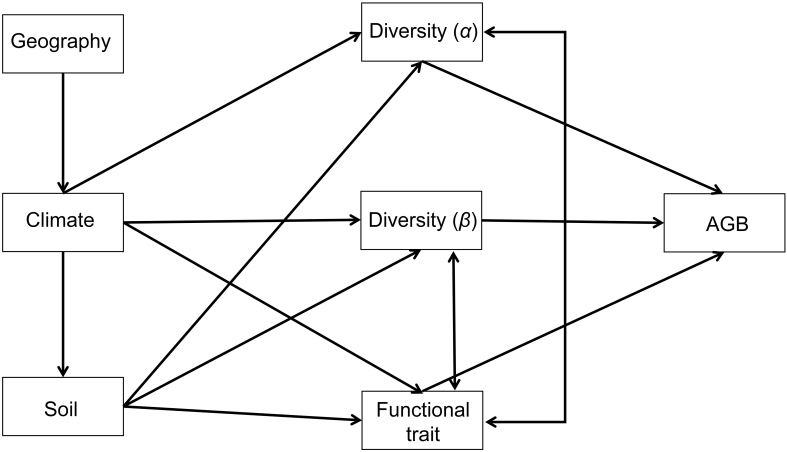


**figs3:**
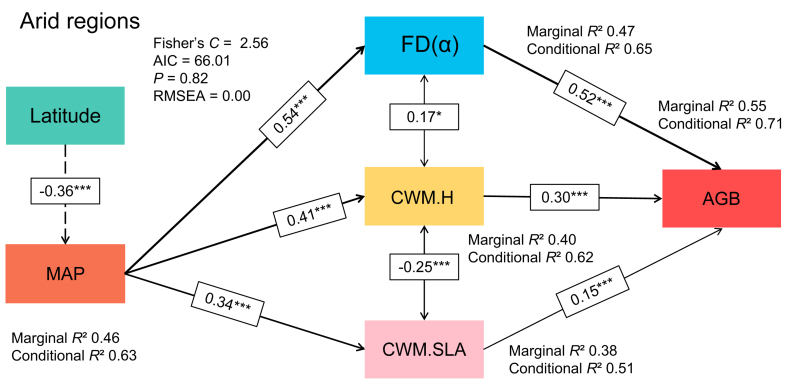


**figs4:**
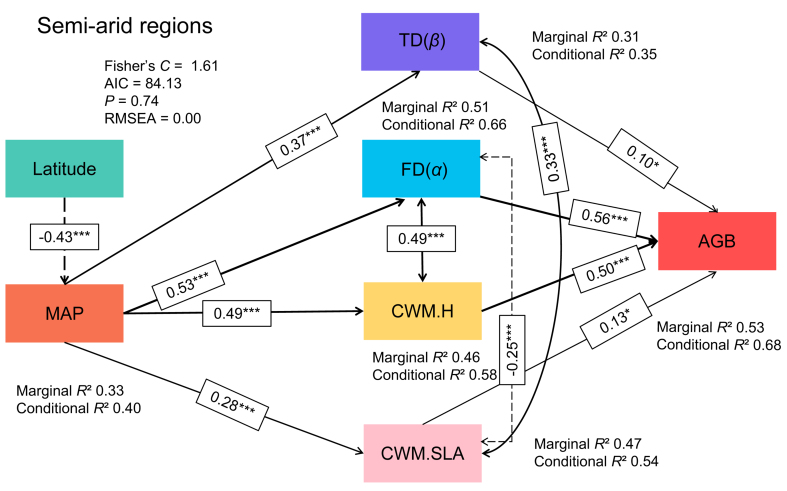


**figs5:**
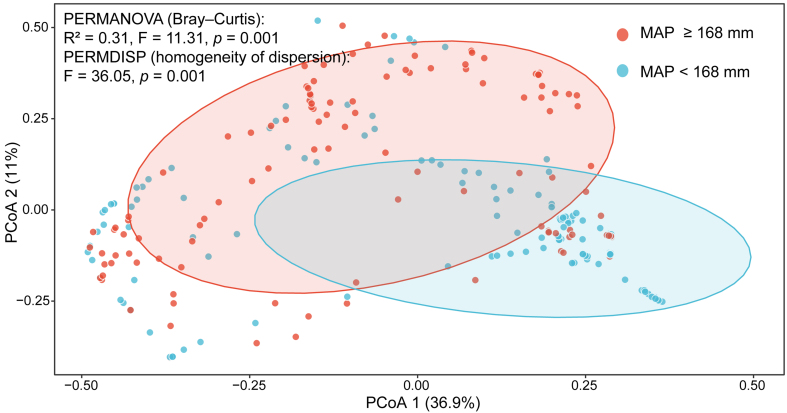


**figs6:**