# Supplementary material for: How does plant diversity drive productivity in China’s drylands: Through complementary or mass ratio effects?
Source: Plant Divers. 2026 May 12;48(4):766–81. doi: 10.1016/j.pld.2026.04.011 (PMC13424705; doi:10.1016/j.pld.2026.04.011)
Supplement: Multimedia component 1 [file mmc1.doc]

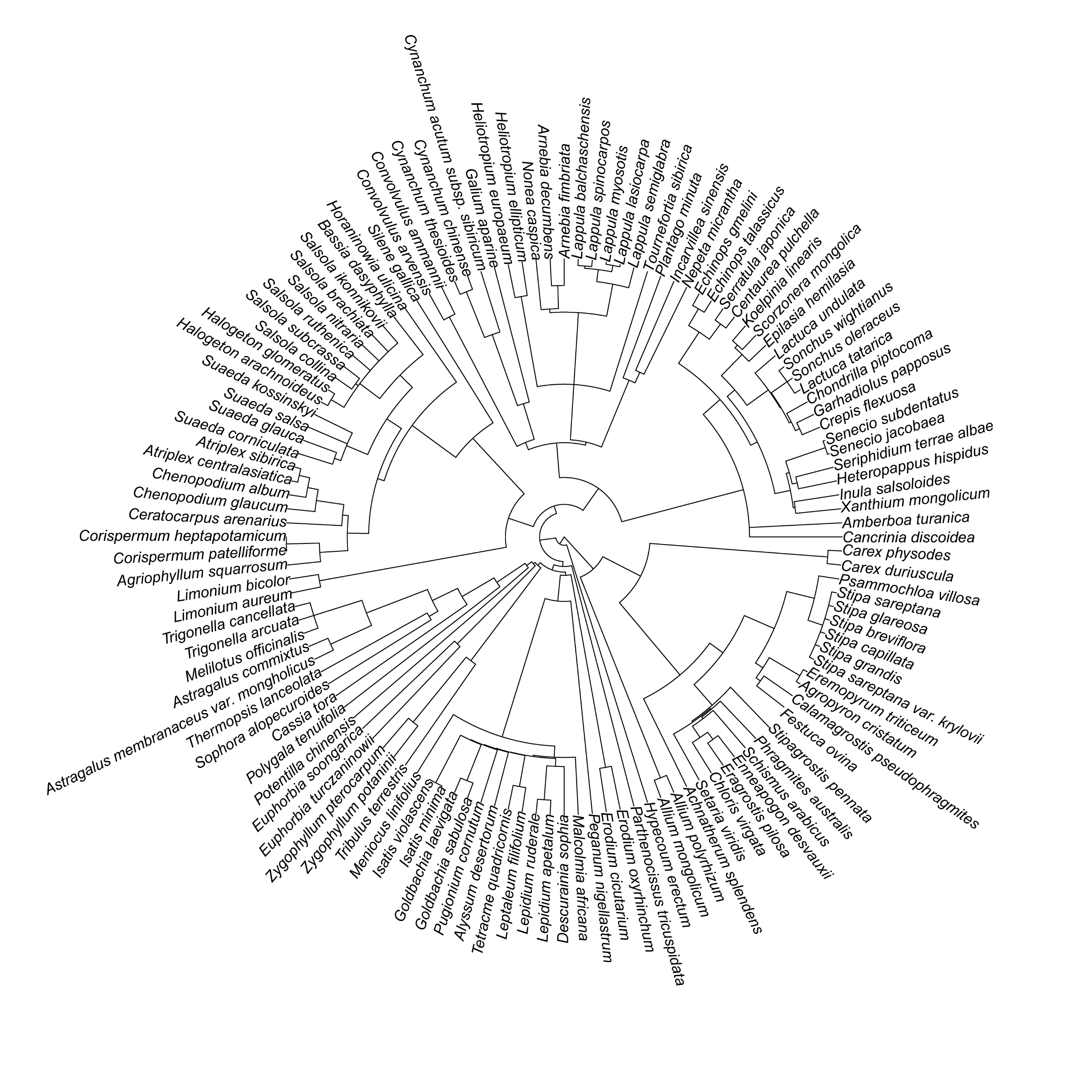


**Fig. S1.** The phylogenetic tree of herbaceous plants found in our study sites

**
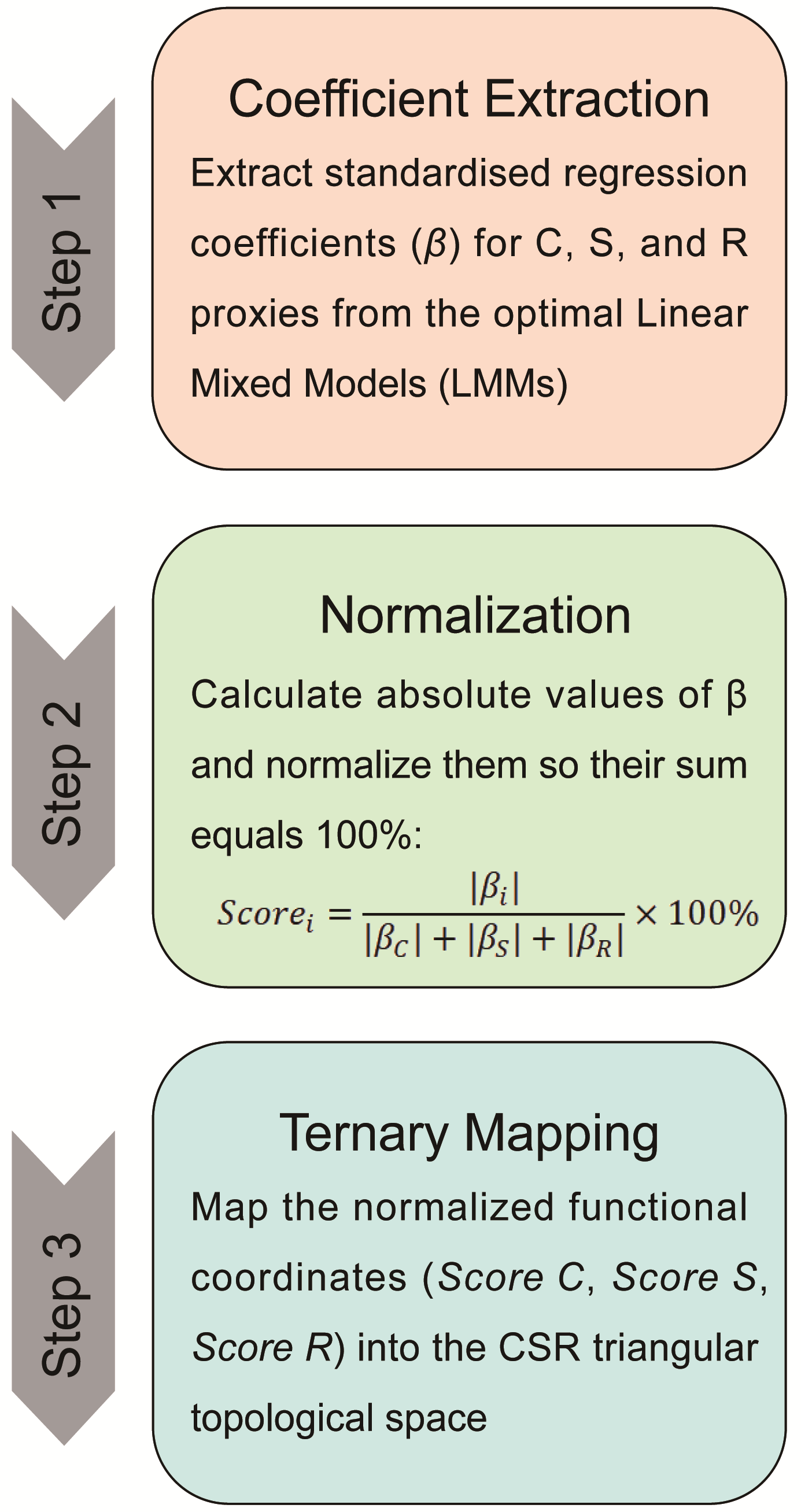
**

**Fig. S2.** Conceptual flowchart of the CSR strategy quantification process. The operational logic maps the translation of statistical model outputs into ecological strategy coordinates through three key steps: (1) extracting standardised β coefficients from LMMs to represent the relative strength of specific ecological drivers; (2) normalizing these absolute values to yield relative percentage coordinates; and (3) projecting these coordinates onto a ternary plot to visualize community functional specialization. C, competition strategy; S, stress tolerance strategy; R, ruderal strategy.

**
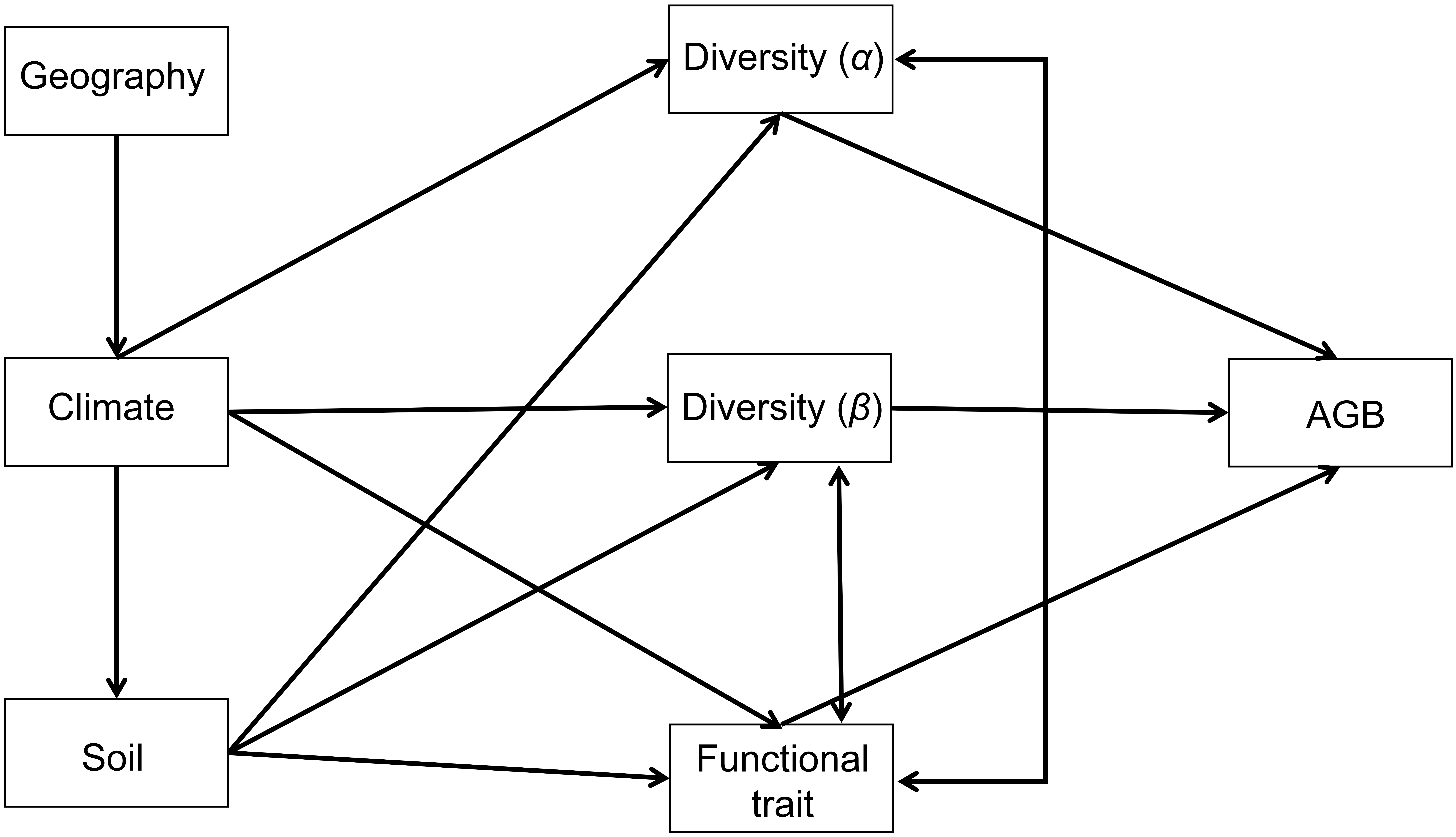
**

**Fig. S3**. The initial a priori Structural Equation Model (SEM) representing the hypothesised causal pathways driving aboveground biomass. The model is structured based on the following ecological hypotheses: abiotic cascade: geographic location is hypothesised to determine regional climatic patterns, which in turn drive soil formation and nutrient status (Path: Geography to Climate to Soil). Environmental filtering: climate and soil conditions are hypothesised to act as primary filters that constrain community functional traits and diversity levels (*α* and *β*) (Path: Climate/Soil to Traits/Diversity). Mass ratio effect: the functional traits of the community are hypothesised to directly regulate AGB, reflecting the influence of dominant species' strategies (Path: Functional trait to AGB). Complementarity and turnover effects: Both local diversity (*α*) and spatial turnover (*β*) are hypothesised to enhance AGB through niche complementarity and spatial insurance effects (Path: Diversity *α*/*β* to AGB). Double-headed arrows represent covariance between variables, indicating non-causal interrelations driven by common underlying processes. Diversity (*α*), alpha diversity; Diversity (*β*), beta diversity; AGB, aboveground biomass. Double-headed arrows indicate covariance trends.

**
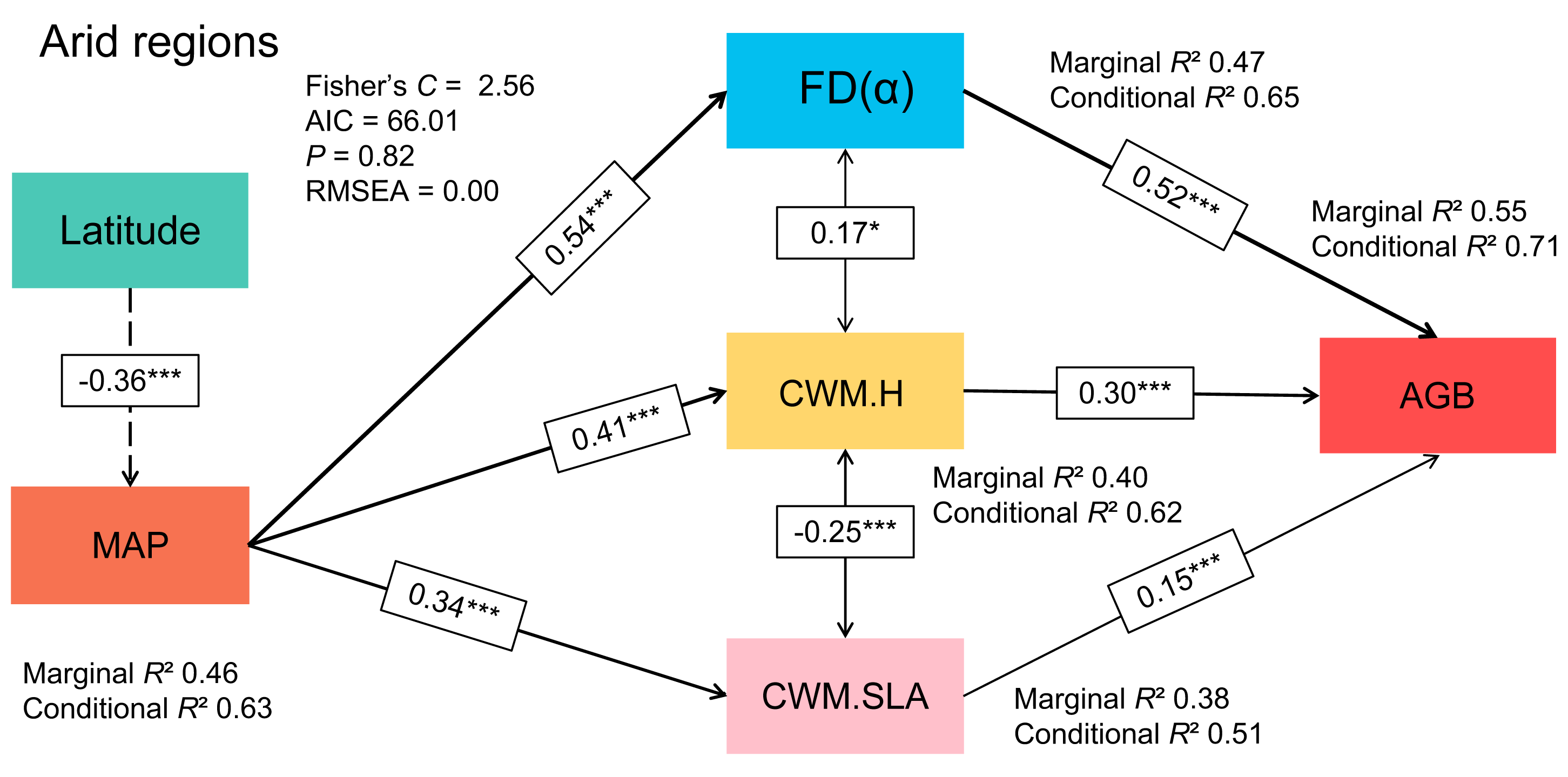
**

**Fig. S4.** Direct and indirect effects of abiotic and biotic variables on plant aboveground biomass in the arid regions. FD (*α*), functional alpha diversity; CWM.H, community weighted mean height; CWM.SLA, community weighted mean specific leaf area; MAP, mean annual precipitation; AGB, aboveground biomass in arid regions. Double-headed arrows indicate covariance trends.

**
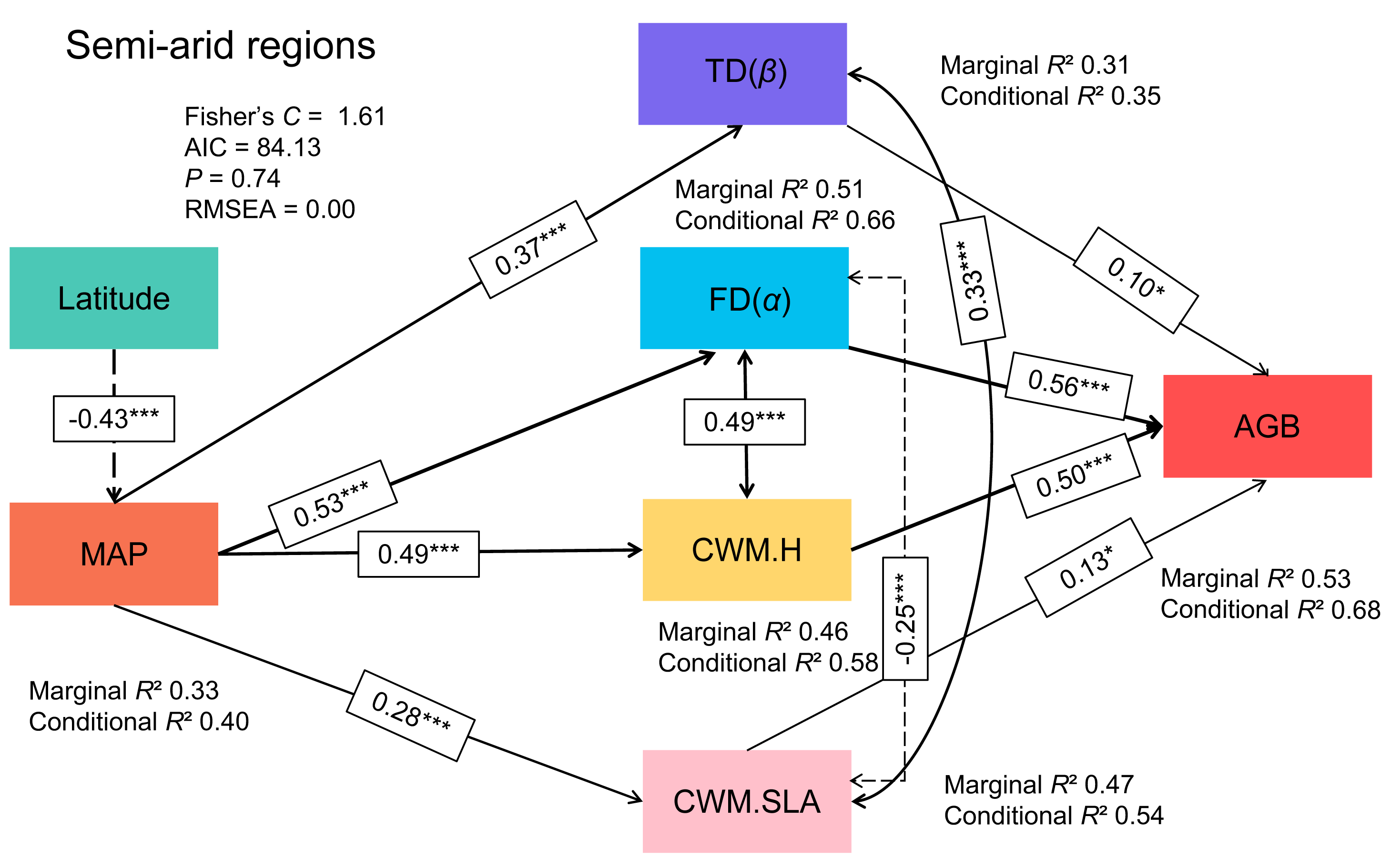
**

**Fig. S5.** Direct and indirect effects of abiotic and biotic variables on plant aboveground biomass in the semi-arid regions. FD (*α*), functional alpha diversity; CWM.H, community weighted mean height; CWM.SLA, community weighted mean specific leaf area; TD (*β*), taxonomic beta diversity; MAP, mean annual precipitation; AGB, aboveground biomass in semi-arid regions. Double-headed arrows indicate covariance trends.

**
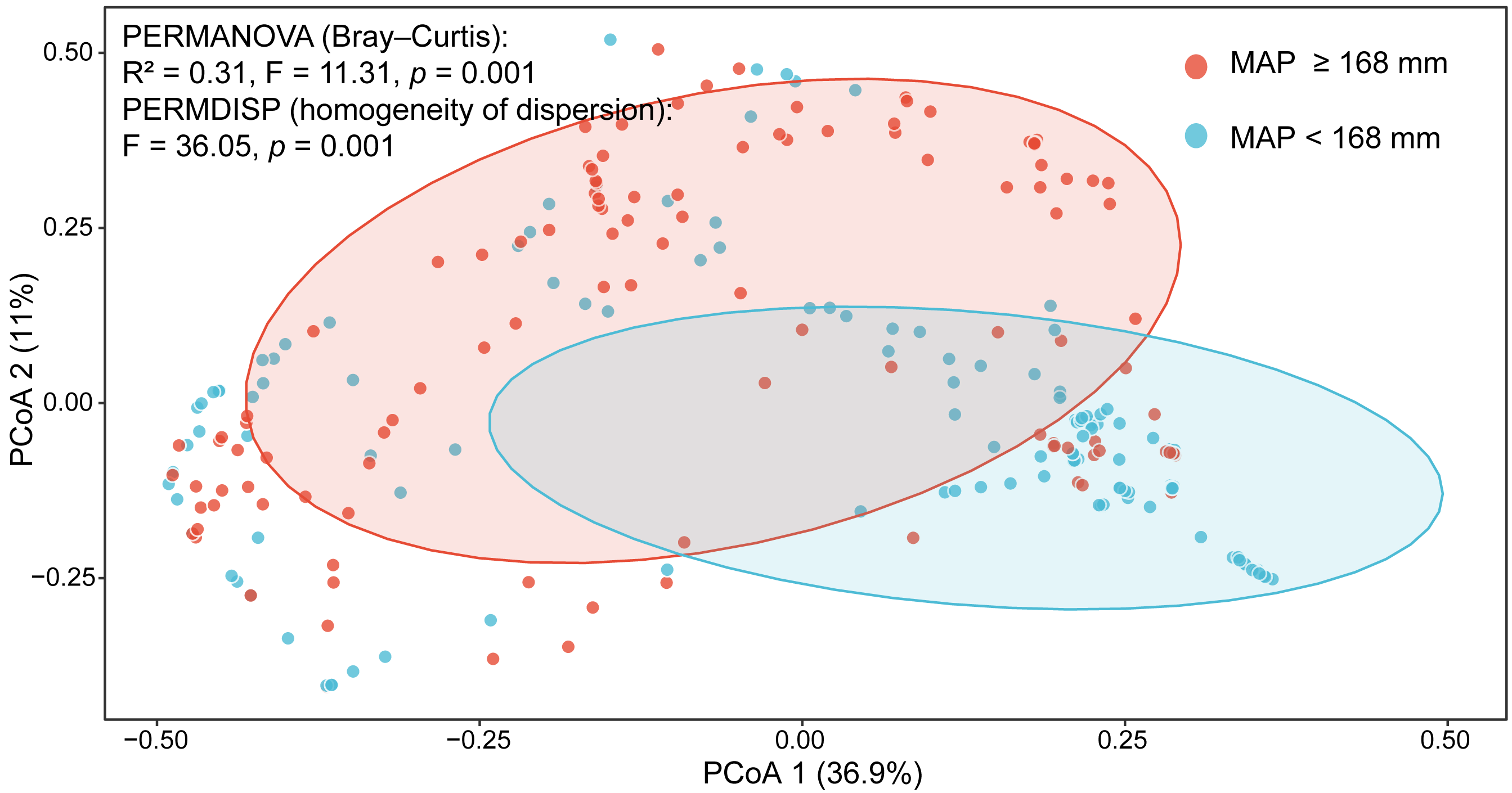
**

**Fig. S6.** Community compositional shift across the MAP = 168 mm threshold. Principal coordinates analysis (PCoA) ordination based on Bray–Curtis dissimilarity calculated from the species abundance matrix across 280 sampling plots. Each point represents one plot, coloured by the precipitation group (MAP < 168 mm vs MAP ≥ 168 mm). Group differences in community composition were tested using PERMANOVA (adonis2; 999 permutations), indicating a significant separation between the two MAP groups (R² = 0.31, F = 11.31, *p* = 0.001). Homogeneity of multivariate dispersions was assessed using PERMDISP (betadisper/permutest; 999 permutations) and was also significant (F = 36.05, *p* = 0.001), suggesting unequal within-group dispersion; therefore, the PERMANOVA result should be interpreted as reflecting both centroid separation and differences in dispersion between groups.

**Table S1** Soil particle size distribution of the study sites in arid and semi-arid regions (mean ± SD).

| Region | Number of sites | 0.05-2mm % | 0.02-0.05mm % | < 0.02 mm % |
| --- | --- | --- | --- | --- |
| Arid regions | 27 | 94.89 ± 4.50 | 4.81 ± 4.11 | 0.25 ± 0.03 |
| Semi-arid regions | 29 | 96.41 ± 3.20 | 3.50 ± 2.99 | 0.13 ± 0.05 |

**Table S2** Ecological effects of functional traits.

| Functional traits | Ecological  Significance | Scale of Inference | Treatment Level | Number of Replicates |
| --- | --- | --- | --- | --- |
| Length-width ratio (L/W) | Influences leaf morphology related to boundary layer thickness, transpiration, and light interception. A higher L/W ratio often aids in dissipating heat and reducing water loss under arid conditions, improving resource use efficiency (Cornelissen et al., 2003; Wright et al., 2004). | Community level | Subplot level | Five |
| Plant height (H) | Relates to vertical light competition, water transport efficiency, and space occupation. Taller species can outcompete shorter ones for light, especially in semi-arid regions where increased resources promote competition (Díaz et al., 2007). | Community level | Subplot level | Five |
| Leaf carbon content (C) | Reflects structural and metabolic investment. Higher C content is often associated with tougher leaves, slower turnover, and adaptation to resource-poor environments, influencing decomposition rates and nutrient cycling (Garnier and Navas, 2012; Villar et al., 2013). | Community level | Subplot level | Five |
| Leaf nitrogen content (N) | Linked to photosynthetic capacity and metabolic rates. Higher N content generally corresponds to increased photosynthetic efficiency, faster growth, and potentially greater responsiveness to resource availability (Reich, 2014; Wright et al., 2004). | Community level | Subplot level | Five |
| Leaf phosphorus content (P) | Leaf P content can influence growth rates, reproductive success, and adaptation to nutrient-limited soils, common in dryland environments (Güsewell, 2004). | Community level | Subplot level | Five |
| Specific leaf area (SLA) | A key trait in the leaf economics spectrum, SLA is the ratio of leaf area to dry mass. Higher SLA indicates thinner leaves adapted for rapid resource acquisition and growth, common in productive conditions, whereas lower SLA reflects thicker, more resource-conservative leaves suitable for stressful, resource-poor environments (Poorter et al., 2009; Wright et al., 2004). | Community level | Subplot level | Five |

Note: The six functional traits quantified in this study were selected to represent multidimensional adaptation strategies of herbaceous plants to arid and semi-arid environments. Height determines light capture ability and competitive hierarchy, which becomes increasingly relevant in semi-arid zones where vegetation cover is denser. SLA is the central trait of the leaf economics spectrum; in drylands, low SLA (high tissue density) is a key indicator of drought tolerance and resource conservation. Leaf shape (L/W) directly influences the leaf boundary layer thickness, affecting transpiration rates and heat exchange—a critical mechanism for avoiding overheating in high-radiation desert environments. Finally, Leaf C, N, and P contents reflect the trade-off between structural investment (high C) and metabolic activity (high N, P), acting as sensitive indicators of nutrient use efficiency in biologically poor desert soils.

**Table S3** The phylogenetic signatures of functional traits in herbaceous plants.

| Functional traits | Blomberg statistic | |
| --- | --- | --- |
|  | K | *p* |
| Length-width ratio (L/W) | 0.05 | 0.26 |
| Plant height (H) | 0.04 | 0.30 |
| Leaf carbon content (C) | 0.03 | 0.48 |
| Leaf nitrogen content (N) | 0.13 | 0.00 |
| Leaf phosphorus content (P) | 0.02 | 0.50 |
| Specific leaf area (SLA) | 0.04 | 0.35 |

Note: K represents Blomberg’s K statistic, which measures the strength of phylogenetic signal in the traits, with values closer to 1 indicating strong phylogenetic conservatism. p indicates the p-value associated with the test for phylogenetic signal, providing statistical evidence for the presence of a phylogenetic pattern.

**Table S4** Results of Model Selection. Akaike information criterion (∆AIC) of the best-selected models for aboveground biomass. FD (*α*), functional alpha diversity; TD (*β*), taxonomic beta diversity; CWM.H, community weighted mean height; Semi-arid regions AGB, aboveground biomass in semi-arid regions; PD (*α*), phylogenetic alpha diversity; TD (*α*), taxonomic alpha diversity; Total regions AGB, aboveground biomass in total regions; Arid regions AGB, aboveground biomass in arid regions; CWM.SLA, community weighted mean specific leaf area; CWM.N, community weighted mean nitrogen content; CWM.P, community weighted mean phosphorus content; PD (*β*), phylogenetic beta diversity; FD (*β*), functional beta diversity; CWM.L/W, community weighted mean leaf length-width ratio; CWM.C, community weighted mean carbon content.

| ID | | Equations | | AICc | ΔAIC | | |
| --- | --- | --- | --- | --- | --- | --- | --- |
| Equation  A1-1 | | Arid regions AGB ~ Latitude + pH + SOC + TN + TP + MAT + MAP + CWM.C + CWM.N + CWM.P + CWM.H + CWM.SLA + CWM.L/W + TD (*α*) + TD (*β*) + FD (*α*) + FD (*β*) + PD (*α*) + PD (*β*) + (1| Site) | | -798.58 | -30.89 | | |
| Equation  A1-2 | | Arid regions AGB ~ Latitude + pH + MAP + CWM.H + CWM.SLA + FD (*α*) + (1| Site) | | -829.47 | 0 | | |
| Equation  A2-1 | | Semi-arid regions AGB ~ Latitude + pH + SOC + TN + TP + MAT + MAP + CWM.C + CWM.N + CWM.P + CWM.H + CWM.SLA + CWM.L/W + TD (*α*) + TD (*β*) + FD (*α*) + FD (*β*) + PD (*α*) + PD (*β*) + (1| Site) | | -579.93 | -85.48 | | |
| Equation A2-2 | Semi-arid regions AGB ~ Latitude + pH + MAP + CWM.H + CWM.SLA + TD (*β*) + FD (*α*) + (1| Site) | | -665.41 | | | 0 |  |
| Equation  A3-1 | | Total regions AGB ~ Latitude + pH + SOC + TN + TP + MAT + MAP + CWM.C + CWM.N + CWM.P + CWM.H + CWM.SLA + CWM.L/W + TD (*α*) + TD (*β*) + FD (*α*) + FD (*β*) + PD (*α*) + PD (*β*) + (1| Site) | | -679.51 | -110.64 | | |
| Equation  A3-2 | | Total regions AGB ~ Latitude + pH + MAP + CWM.H + CWM.SLA + TD (*α*) + FD (*α*) + FD (*β*) + (1| Site) | | -790.15 | 0 | | |

**Table S5** Variance inflation factor (VIF) collinearity test for predictor. SWC, soil water content; SOC, Soil organic carbon; TN, soil total nitrogen; TP, soil total phosphorus; AI, aridity index; MAT, annual mean temperature; MAP, annual mean precipitation; CWM.C, community weighted mean carbon content; CWM.N, community weighted mean nitrogen content; CWM.P, community weighted mean phosphorus content; CWM.L/W, community weighted mean leaf length-width ratio; CWM.H, community weighted mean height; CWM.SLA, community weighted mean specific leaf area; TD (*β*), taxonomic beta diversity; TD (*α*), taxonomic alpha diversity; FD (*α*), functional alpha diversity; FD (*β*), functional beta diversity; PD (*α*), phylogenetic alpha diversity; PD (*β*), phylogenetic beta diversity.

| Index | VIF- Equations 1 | VIF- Equations 2 | VIF- Equations 3 |
| --- | --- | --- | --- |
| Latitude | 2.77 | 2.17 | 1.01 |
| longitude | 13.05 | 13.41 | 12.25 |
| elevation | 16.44 | 14.64 | 13.01 |
| SWC | 18.29 | 18.12 | 17.25 |
| pH | 1.02 | 1.01 | 1.00 |
| SOC | 2.65 | 2.63 | 2.55 |
| TN | 5.78 | 5.53 | 5.34 |
| TP | 6.29 | 6.66 | 6.49 |
| AI | 19.28 | 18.94 | 17.99 |
| MAT | 1.34 | 1.28 | 1.19 |
| MAP | 7.99 | 6.06 | 5.34 |
| CWM.C | 2.21 | 2.17 | 2.05 |
| CWM.N | 6.38 | 6.24 | 6.00 |
| CWM.P | 8.24 | 8.28 | 8.19 |
| CWM.L/W | 4.60 | 3.57 | 3.59 |
| CWM.H | 5.27 | 5.33 | 5.47 |
| CWM.SLA | 2.45 | 3.57 | 2.08 |
| TD (*α*) | 2.24 | 2.07 | 2.06 |
| TD (*β*) | 5.88 | 5.79 | 5.71 |
| FD (*α*) | 3.62 | 5.01 | 2.94 |
| FD (*β*) | 2.79 | 2.74 | 2.13 |
| PD (*α*) | 1.99 | 1.99 | 1.25 |
| PD (*β*) | 3.84 | 3.55 | 3.43 |

**Table S6** Sensitivity analysis of precipitation thresholds to variations in rolling-window size (*k*). CWM.H, community weighted mean height; CWM.SLA, community weighted mean specific leaf area; FD (*α*), functional alpha diversity.

| Variable | *k* | ΔAIC fixed | *P* shift | Best breakpoint (mm) | 95% CI (mm) | 168 mm in CI? |
| --- | --- | --- | --- | --- | --- | --- |
| FD (*α*) | 6 | 85.2 | *P < 0.05* | 156.5 | 153.2–178.5 | Yes |
| FD (*α*) | 8 | 115.8 | *P < 0.01* | 157.8 | 154.5–172.6 | Yes |
| FD (*α*) | 10 | 139.6 | *p < 0.001* | 159 | 152.1–169.5 | Yes |
| FD (*α*) | 12 | 105.4 | *p < 0.05* | 162.3 | 153.8–175.4 | Yes |
| FD (*α*) | 14 | 72.1 | *p < 0.05* | 164.5 | 155.3–182.6 | Yes |
| CWM.H | 6 | 412.5 | *p < 0.05* | 163.4 | 158.2–175.4 | Yes |
| CWM.H | 8 | 580.3 | *p < 0.01* | 166.1 | 163.5–171.2 | Yes |
| CWM.H | 10 | 619.9 | *p < 0.001* | 168 | 167.8–169.7 | Yes |
| CWM.H | 12 | 550.8 | *p < 0.001* | 170.2 | 165.5–174.8 | Yes |
| CWM.H | 14 | 425.1 | *p < 0.05* | 173.5 | 160.1–179.2 | Yes |
| CWM.SLA | 6 | 289.4 | *p < 0.05* | 160.8 | 155.5–178.0 | Yes |
| CWM.SLA | 8 | 395.2 | *p < 0.05* | 163.5 | 160.2–175.5 | Yes |
| CWM.SLA | 10 | 444.7 | *p < 0.001* | 165 | 163.3–171.4 | Yes |
| CWM.SLA | 12 | 380.6 | *p < 0.01* | 167.9 | 159.8–176.2 | Yes |
| CWM.SLA | 14 | 275.3 | *p < 0.05* | 171.4 | 156.4–180.5 | Yes |

**Table S7.** Standardised regression coefficients for diversity and environmental factors at different scales.

|  | Factors | Estimate | Std. Error | *t*-value | *p* |
| --- | --- | --- | --- | --- | --- |
| Arid regions (Equation A1-2) | Latitude | -0.0924 | 0.0152 | -6.072 | *p < 0.001* |
|  | pH | 0.0492 | 0.0182 | 2.701 | *p < 0.01* |
|  | MAP | 0.4230 | 0.0231 | 18.264 | *p < 0.001* |
|  | CWM.H | 0.2878 | 0.0325 | 8.836 | *p < 0.001* |
|  | CWM.SLA | 0.1893 | 0.0346 | 5.253 | *p < 0.001* |
|  | FD (*α*) | 0.4387 | 0.0398 | 11.000 | *p < 0.001* |
| Semi-arid regions  (Equation A2-2) | Latitude | -0.2495 | 0.0395 | -6.315 | *p < 0.001* |
|  | pH | 0.1050 | 0.0256 | 4.091 | *p < 0.001* |
|  | MAP | 0.1696 | 0.0451 | 3.758 | *p < 0.001* |
|  | CWM.H | 0.3106 | 0.0501 | 6.2 | *p < 0.001* |
|  | CWM.SLA | 0.1173 | 0.0196 | 5.965 | *p < 0.001* |
|  | TD (*β*) | 0.2412 | 0.0388 | 6.211 | *p < 0.001* |
|  | FD (*α*) | 0.4582 | 0.0592 | 7.731 | *p < 0.001* |
| Total regions  (Equation A3-2) | Latitude | -0.2015 | 0.0162 | -12.426 | *p < 0.001* |
|  | pH | 0.0318 | 0.0192 | 1.655 | *p < 0.05* |
|  | MAP | 0.1951 | 0.0269 | 7.23 | *p < 0.001* |
|  | CWM.H | 0.7354 | 0.0577 | 12.738 | *p < 0.001* |
|  | CWM.SLA | 0.2756 | 0.0362 | 7.598 | *p < 0.001* |
|  | TD (*α*) | 0.0118 | 0.0038 | 3.09 | *p < 0.05* |
|  | FD (*α*) | 0.1338 | 0.0544 | 2.456 | *p < 0.05* |
|  | FD (*β*) | 0.0919 | 0.0376 | 2.446 | *p < 0.05* |

**Text S1.** Rationale for retaining the first principal component (PC1) in CSR strategy quantification

In this study, Principal Component Analysis (PCA) was utilised to synthesise multiple intercorrelated indicators into composite proxies for the respective CSR strategies. We exclusively retained the first principal component (PC1) for downstream ternary coordinate calculations. This methodological choice was based on two core ecological and statistical rationales:

1. Capturing the Primary Coordinated Axis: In functional ecology, the dominant axis of a PCA (PC1) typically captures the primary syndrome of coordinated trait variation, such as the overarching acquisitive-conservative continuum (Wright et al., 2004; Díaz et al., 2016). In our dataset, PC1 accounted for largest proportion of the variance (44.8%), representing a robust and dominant ecological trade-off axis. While incorporating subsequent orthogonal components (PC2, 13.9%) might mathematically increase the total variance explained, these secondary axes often represent independent, uncorrelated ecological gradients or localised noise that do not coherently align with the unidimensional theoretical definition of a specific CSR strategy, such as competitive dominance (Grime, 1977).

2. Model Parsimony and Clarity: Retaining only PC1 effectively eliminates multicollinearity among raw indicators while mathematically distilling the strongest, most coherent ecological signal. A weighted average of multiple PCs could dilute this primary signal by blending orthogonal dimensions, thereby obscuring the clear functional trajectory of communities mapped in the CSR ternary space (Díaz et al., 2016). Therefore, PC1 serves as the most robust, parsimonious, and ecologically interpretable proxy for our focal assembly mechanisms.

**References**

Cornelissen, J.H.C., Lavorel, S., Garnier, E., et al., 2003. A handbook of protocols for standardised and easy measurement of plant functional traits worldwide. Aust. J. Bot. 51, 335–380. <https://doi.org/10.1071/BT02124>.

Díaz, S., Kattge, J., Cornelissen, J.H.C., et al., 2016. The global spectrum of plant form and function. Nature 529, 167–171. <https://doi.org/10.1038/nature16489>.

Díaz, S., Lavorel, S., de Bello, F., et al., 2007. Incorporating plant functional diversity effects in ecosystem service assessments. Proc. Natl. Acad. Sci. U. S. A. 104, 20684–20689. <https://doi.org/10.1073/pnas.0704716104>.

Garnier, E., Navas, M.-L., 2012. A trait-based approach to comparative functional plant ecology: concepts, methods and applications for agroecology. A review. Agron. Sustain. Dev. 32, 365–399. <https://doi.org/10.1007/s13593-011-0036-y>.

Grime, J.P., 1977. Evidence for the existence of three primary strategies in plants and its relevance to ecological and evolutionary theory. Am. Nat. 111, 1169–1194. <https://doi.org/10.1086/283244>.

Güsewell, S., 2004. N:P ratios in terrestrial plants: variation and functional significance. New Phytol. 164, 243–266. <https://doi.org/10.1111/j.1469-8137.2004.01192.x>.

Poorter, H., Niinemets, Ü., Poorter, L., et al., 2009. Causes and consequences of variation in leaf mass per area (LMA): a meta-analysis. New Phytol. 182, 565–588. <https://doi.org/10.1111/j.1469-8137.2009.02830.x>.

Reich, P.B., 2014. The world-wide ‘fast–slow’ plant economics spectrum: a traits manifesto. J. Ecol. 102, 275–301. <https://doi.org/10.1111/1365-2745.12211>.

Villar, R., Ruiz-Robleto, J., Ubera, J.L., et al., 2013. Exploring variation in leaf mass per area (LMA) from leaf to cell: an anatomical analysis of 26 woody species. Am. J. Bot. 100, 1969–1980. https://doi.org/10.3732/ajb.1200562.

Westoby, M., 1998. A leaf-height-seed (LHS) plant ecology strategy scheme. Plant Soil 199, 213–227. <https://doi.org/10.1023/A:1004327224729>.

Wright, I.J., Reich, P.B., Westoby, M., et al., 2004. The worldwide leaf economics spectrum. Nature 428, 821–827. <https://doi.org/10.1038/nature02403>.
